# Integrated analysis to reveal potential therapeutic targets and prognostic biomarkers of skin cutaneous melanoma

**DOI:** 10.3389/fimmu.2022.914108

**Published:** 2022-08-11

**Authors:** Xuezhi Zhou, Rong Rong, Siqi Xiong, Weitao Song, Dan Ji, Xiaobo Xia

**Affiliations:** ^1^ Eye Center of Xiangya Hospital, Central South University, Changsha, China; ^2^ Hunan Key Laboratory of Ophthalmology, Changsha, China; ^3^ National Clinical Research Center for Geriatric Diseases (Xiangya Hospital), Changsha, China

**Keywords:** immune infiltration, skin cutaneous melanoma, long non-coding RNA, microRNA, mRNA, DNA methylation

## Abstract

Skin cutaneous melanoma (SKCM) is a malignant tumor with high mortality rate in human, and its occurrence and development are jointly regulated by genes and the environment. However, the specific pathogenesis of SKCM is not completely understood. In recent years, an increasing number of studies have reported the important role of competing endogenous RNA (ceRNA) regulatory networks in various tumors; however, the complexity and specific biological effects of the ceRNA regulatory network of SKCM remain unclear. In the present study, we obtained a ceRNA regulatory network of long non-coding RNAs, microRNAs, and mRNAs related to the phosphatase and tensin homolog (*PTEN*) in SKCM and identified the potential diagnostic and prognostic markers related to SKCM. We extracted the above three types of RNA involved in SKCM from The Cancer Genome Atlas database. Through bioinformatics analysis, the *OIP5-AS1*-hsa-miR-186-5p/hsa-miR-616-3p/hsa-miR-135a-5p/hsa-miR-23b-3p/hsa-miR-374b-5p-*PTPRC*/*IL7R*/*CD69* and *MALAT1*-hsa-miR-135a-5p/hsa-miR-23b-3p/hsa-miR-374b-5p-*IL7R*/*CD69* ceRNA networks were found to be related to the prognosis of SKCM. Finally, we determined the *OIP5-AS1-PTPRC*/*IL7R*/*CD69* and *MALAT1-IL7R*/*CD69* axes in ceRNA as a clinical prognostic model using correlation and Cox regression analyses. Additionally, we explored the possible role of these two axes in affecting gene expression and immune microenvironment changes and the occurrence and development of SKCM through methylation and immune infiltration analyses. In summary, the ceRNA-based *OIP5-AS1-PTPRC*/*IL7R*/*CD69* and *MALAT1-IL7R*/*CD69* axes may be a novel and important approach for the diagnosis and prognosis of SKCM.

## Introduction

Although melanoma accounts for only 5% of all skin cancers, it results in 75% of skin cancer deaths and is highly aggressive and fatal (1). The prognosis of skin cutaneous melanoma (SKCM) is poor without surgical resection (2). SKCM causes 55,500 deaths every year, and its global incidence is increasing every year at a faster rate than that of other cancers. The morbidity and mortality rates of this disease vary greatly worldwide, mainly depending on the availability of early detection and primary care (3). Although there is still no satisfactory clinical treatment for aggressive skin malignant tumors, the approval of tyrosine kinase and immune checkpoint inhibitors has led to revolutionary changes in the treatment of melanoma as these inhibitors have a considerable impact on melanoma prognosis. However, the ensuing problem of drug resistance poses further challenges to current clinical management. Moreover, melanoma cells closely interact with the tumor microenvironment and immune system, and non-coding RNAs have recently been reported to play key roles in the occurrence and development of tumors (4). This information can help determining changes in tumor treatment targets and strategies. Therefore, the discovery of new diagnostic and therapeutic targets and prognostic indicators for SKCM is essential for advancing its clinical diagnosis, treatment, and prognosis.

Long non-coding RNAs (lncRNAs) are non-coding RNAs with a length greater than 200 nucleotides (5) that are widely distributed in human organs and tissues. An increasing number of studies has shown that lncRNAs are involved in the progression of a variety of cancers and generally function as tumor diagnostic and prognostic markers (6). Most reports have demonstrated that lncRNAs exert its biological role *via* the competing endogenous RNAs (ceRNAs) mechanism (7). The ceRNA hypothesis suggests a new mechanism of interaction between the different types of RNAs by which microRNAs (miRNAs) bind to target mRNAs, inhibiting their translation or leading to their degradation; ceRNAs participate in the post-transcriptional regulation of gene expression by competitively binding to miRNAs (7). These single-stranded non-coding RNAs with a length of 21–23 nucleotides (8) have been particularly investigated regarding their role in tumor formation (9). miRNAs can function directly by interacting with mRNAs or other non-coding RNAs to indirectly affect mRNA expression (10). Many studies have focused on the ceRNA network related to lncRNAs in SKCM (11), but the pathogenesis of SKCM is intricate, and therefore, such studies are limited. Many other studies have only focused on lncRNA-miRNA interactions. However, in complex disease environments, one lncRNA can often bind to multiple miRNAs or multiple lncRNAs can bind to one miRNA (12), forming a complex network, which remains unclear. Interactions between RNAs in SKCM can be understood through ceRNA networks, which can reveal the pathogenesis of SKCM comprehensively and provide clinical diagnostic and/or prognostic markers for SKCM.

Phosphatase and tensin homolog (*PTEN*) is the most widely studied tumor suppressor gene regarding ceRNA and the only tumor suppressor gene with dual phosphatase activity (lipids and proteins) specificities identified to date. *PTEN* is also involved in the regulation of multiple intracellular signal transduction pathways and indispensable for preventing the occurrence and development of many cancers, as its deletion or mutation allows malignant cells to grow and metastasize, leading to cancer progression (13). Accordingly, *PTEN* is generally mutated in tumor tissues, including SKCM (14). Its low expression or inactivation can lead to SKCM tumor cell proliferation and invasion, and the low expression level of *PTEN* is directly related to SKCM tumor size, severity, metastasis, and marker levels (15, 16).

Research on SKCM-related disease models and potential prognostic biomarkers has mainly focused on the impact of single genes (17) and on the construction and prediction of ceRNA networks (11). However, the prognosis of SKCM in patients varies greatly because it is related to individual differences and to the complexity of molecular pathological changes in SKCM. Single and broad-use prognostic indicators of SKCM have been proposed in previous studies but there is a lack of in-depth and precise investigation on the distinct gene regulatory networks of SKCM. Although *PTEN* is widely expressed in SKCM and its low expression usually affects the prognosis SKCM patients, the ceRNA network associated with *PTEN* in SKCM has not yet been identified. Therefore, the present study aimed to identify the expression profile of the lncRNA-related ceRNA network in *PTEN*-expressing SKCM.

The lncRNA-related ceRNA network analysis for the *PTEN* gene based on The Cancer Genome Atlas (TCGA) public database allowed classifying SKCM tissues as PTEN^high^ and PTEN^low^ according to their high or low expression of *PTEN*, respectively. These two groups were compared and analyzed, as well as cancer tissues and normal skin tissues, considering three types of RNA: lncRNA, miRNA, and mRNA. Through function enrichment analysis of related genes, the possible biological functions and roles of the ceRNA network in SKCM were described. Two main ceRNA axes were constructed, and the relevant biological processes and molecular pathways of these main networks in SKCM were obtained through correlation, survival, and gene ontology (GO) and Kyoto Encyclopedia of Genes and Genomes (KEGG) analyses. These led to the determination of the essential role of *OIP5-AS1-PTPRC*/*IL7R*/*CD69* and *MALAT1-IL7R*/*CD69* in SKCM. Furthermore, the methylation and immune infiltration analyses of *PTPRC*, *IL7R*, and *CD69* revealed the potential biological functions of these three RNAs in SKCM.

## Materials and methods

### Data collection and processing

**Figure 1:**
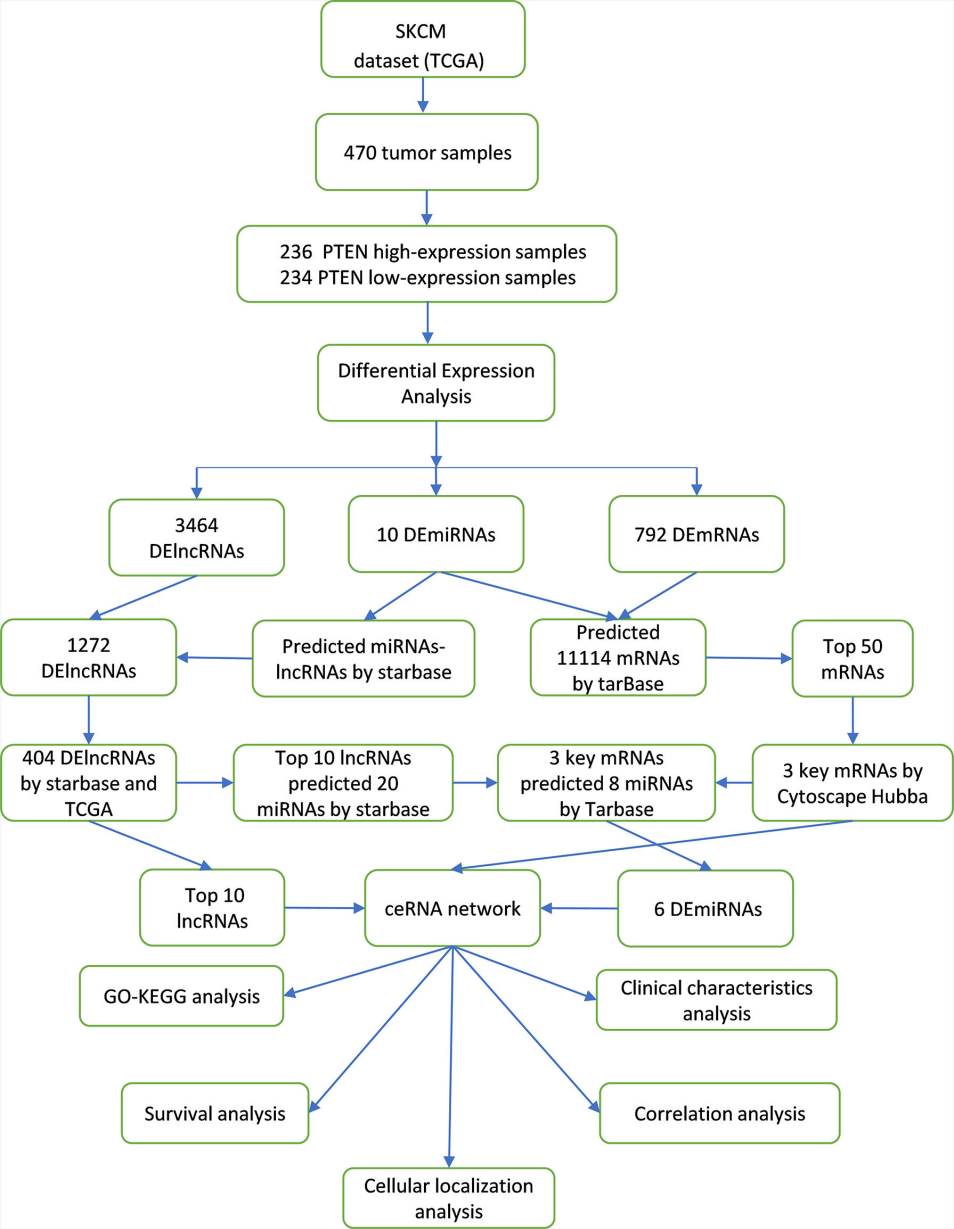
Construction and analysis of the ceRNA network in SKCM.

A scheme of our research design is shown in **Figure 1**. The fragments per kilobase of transcripts per million reads mapped data from high-throughput RNA sequencing (RNA-Seq) were downloaded from the TCGA (https://portal.gdc.cancer.gov/; version 1.28.0) SKCM project and then converted into transcripts per million data followed by log2 transformation for correlation analysis. For performing differential analysis, the high-throughput RNA-Seq-counts data from the TCGA SKCM project was downloaded and normalized using the DESeq2 package (version 1.26.0) (18); the ggplot2 package (version 3.3.3) (19) of R (https://www.r-project.org) was then used to visualize the differential analysis results. The data included SKCM lncRNA, mRNA, and miRNA sequences. The reference genome included in the TCGA database was from the GENCODE database (GRCh38) (20). To further validate our results, the gene expression datasets of GSE3189 (including 45 SKCM samples and seven normal skin samples; platform GPL96) were downloaded from the Gene Expression Omnibus (GEO) database (https://www.ncbi.nlm.nih.gov/geo/) (21). The expression levels of differentially expressed (DE) mRNAs (*IL7R*, *CD69*, and *PTPRC*) in different human tissues were verified using the Cancer Cell Line Encyclopedia (CCLE; https://sites.broadinstitute.org/ccle/), and data preprocessing and normalization were performed as described previously (22). The expression levels of *PTEN* and related genes in SKCM and normal skin tissues were verified using the Human Protein Atlas (HPA) database (https://www.proteinatlas.org/; version 21.1), and data preprocessing and normalization were performed as described previously (23). Based on HPA data, HPA031335 was selected as the anti-*PTEN* antibody and purchased from Sigma-Aldrich (St Louis, MO, USA). The mutation status of *PTEN* and related genes was obtained from the cBioPortal database (https://www.cbioportal.org/; version 4.1.13) (24). The data were retrieved on 16 August 2021.

### Differential expression screening

When analyzing the expression of *PTEN* in the SKCM dataset, to determine high- and low-expression *PTEN* groups, and when performing the differential expression analysis between SKCM and normal skin tissues, lncRNAs, miRNAs, and mRNAs were considered as DE at |log fold-change (FC)| > 0.5 and adjusted p < 0.05. Volcano plots and heatmap clustering of the DERNAs (including DElncRNAs, DEmiRNAs, and DEmRNAs) were visualized using the ggplot2 package of R. Cytoscape (https://cytoscape.org; version 3.8.2) (25) was used to perform GO/KEGG analyses and visualize differentially expressed genes (DEGs).

### Construction of the ceRNA network in SKCM

The expression level of mRNA is regulated by miRNA. There is a competitive relationship between lncRNAs and miRNAs, which can regulate mRNA expression through sponge adsorption. We used the starBase (https://starbase.sysu.edu.cn/index.php) (26) database to predict the lncRNA-miRNA interactions and obtained all lncRNAs that could interact with the different miRNAs. By intersecting the 1,272 predicted lncRNAs with the 3,464 DElncRNAs resulting from TCGA database query, 404 lncRNAs were obtained by using VennDiagram package (version 1.7.3) (27) of R and 20 associated miRNAs were predicted using starBase and the top ten most divergent lncRNAs among the 404 lncRNAs. The DEmiRNAs between in the PTEN^high^ and PTEN^low^ groups were used to predict the corresponding DEmRNAs using the TarBase database (https://dianalab.e-ce.uth.gr/html/diana/web/index.php?r=tarbasev8; version 8) (28) and intersected with DEmRNAs analyzed using TCGA data, 266 mRNAs were obtained by using VennDiagram package of R. Then, we selected the top 50 DEmRNAs and analyzed them in the CytoHubba plugin (29) of the Cytoscape software to obtain the top three core mRNAs with the most significant correlations. Eight miRNAs corresponding to these three mRNAs were predicted using the TarBase database (28). Based on the intersection of the 20 miRNAs predicted by the top ten lncRNAs and the miRNAs predicted by the mRNAs, six miRNAs were obtained by using VennDiagram package of R. These lncRNAs, mRNAs, and miRNAs were imported into the CytoHubba plugin of Cytoscape to obtain the final two lncRNAs, five miRNAs, and three mRNAs, which were screened to construct the ceRNA network based on the overlapping miRNAs. These were selected by comparing all predicted miRNAs with the DEmiRNAs using the VennDiagram package (version 1.7.3) of R. The sequence information of lncRNAs was acquired from LNCipedia (https://lncipedia.org/; version 5.2) (30). The distribution of the screened DElncRNAs in the cell was obtained from the lncLocator database (http://www.csbio.sjtu.edu.cn/bioinf/lncLocator/; version 2.0) (31).

### Enrichment analyses of DEGs

To analyze the mechanisms through which the main DEGs play a role in the occurrence of SKCM, we conducted enrichment analyses of DEGs. First, we analyzed the DEmRNAs in the ceRNA network using the Metascape database (https://metascape.org/gp/index.html#/main/step1; version 3.5) (32) and STRING database (https://cn.string-db.org/; version 11.5) (33). Then, we performed single-gene GO enrichment and KEGG analyses on *OIP5-AS1*, *MALAT1*, *PTPRC*, *IL7R*, and *CD69* in the ceRNA network by using DESeq2 package and clusterProfiler package (version 3.14.3) of R, and the analysis results were visualized using the ggplot2 package of R.

### Relationship between the ceRNA network and the survival rate of SKCM patients

The fragments per kilobase of transcripts per million reads mapped data from high-throughput RNA-Seq were downloaded from the TCGA (https://portal.gdc.cancer.gov/; version 1.28.0) SKCM project and then converted into transcripts per million data followed by log2 transformation. Kaplan–Meier analysis of survival data was performed by the survival package (version 3.2-10), and the analysis results were visualized by the survminer package (version 0.4.9). The Logistic regression analysis model was constructed by the basic package of R, where the independent variable was OIP5-AS1, the low expression of OIP5-AS1 was used as the reference, and the dependent variables were TNM stage, Age, Gender, BMI, Pathologic stage, and Breslow depth. IL7R, PTPRC, CD69 and MALAT1 were analyzed in the same way as OIP5-AS1. We analyzed the survival rate of SKCM patients by Cox regression by using survival package (3.2-10 version), considering the DElncRNAs, DEmiRNAs, and DEmRNAs in the ceRNA network, and used these data to determine important biological markers related to the prognosis of SKCM.

### *CD69*, *IL7R*, and *PTPRC* methylation and expression analysis

DNA methylation can cause changes in chromatin structure, DNA conformation, DNA stability, and the way DNA interacts with proteins, thereby regulating gene expression and affecting the characteristics of tumor cells. We used the DiseaseMeth (http://biobigdata.hrbmu.edu.cn/diseasemeth/; version 2.0) (34) and The University of Alabama at Birmingham Cancer data analysis portal (UALCAN, http://ualcan.path.uab.edu) (35) databases to evaluate the methylation levels of genes *CD69*, *IL7R*, and *PTPRC* in tumor and para-carcinoma tissues of SKCM patients. Simultaneously, the correlation between their methylation levels and important genes was analyzed by MEXPRESS (https://mexpress.be; new version) (36). Finally, MethSurv (https://biit.cs.ut.ee/methsurv/) (37) was used to perform a multivariate survival analysis to evaluate the dispersion of different CpG islands. The version numbers of UALCAN database and MethSurv database were not found on the official website.

### The correlation between immune infiltration and CD69, *IL7R*, and *PTPRC* in SKCM

The correlation between immune infiltration and *CD69*, *IL7R*, and *PTPRC* genes and selected variables, was analyzed using TIMER (http://timer.cistrome.org/; version 2.0) (38). The relationship between *CD69*, *IL7R*, and *PTPRC*, their mutations and prognosis, with SKCM immune-infiltrating B cells, CD4+ T cells, CD8+ T cells, neutrophils, macrophages, and dendritic cells were also analyzed using TIMER (38).

### Statistical methods

SPSS (version 22.0) was used for data analysis. The results are presented as the median and 95% confidence interval. The Mann-Whitney test and independent *t*-test were used to evaluate the differences between any two groups of data. One-way analysis of variance and the Kruskal-Wallis and chi-square tests were utilized to evaluate the differences among different groups of data. Statistical differences were considered at p < 0.05.

## Results

### The expression of *PTEN* in SKCM and its influence on SKCM prognosis

**Figure 2:**
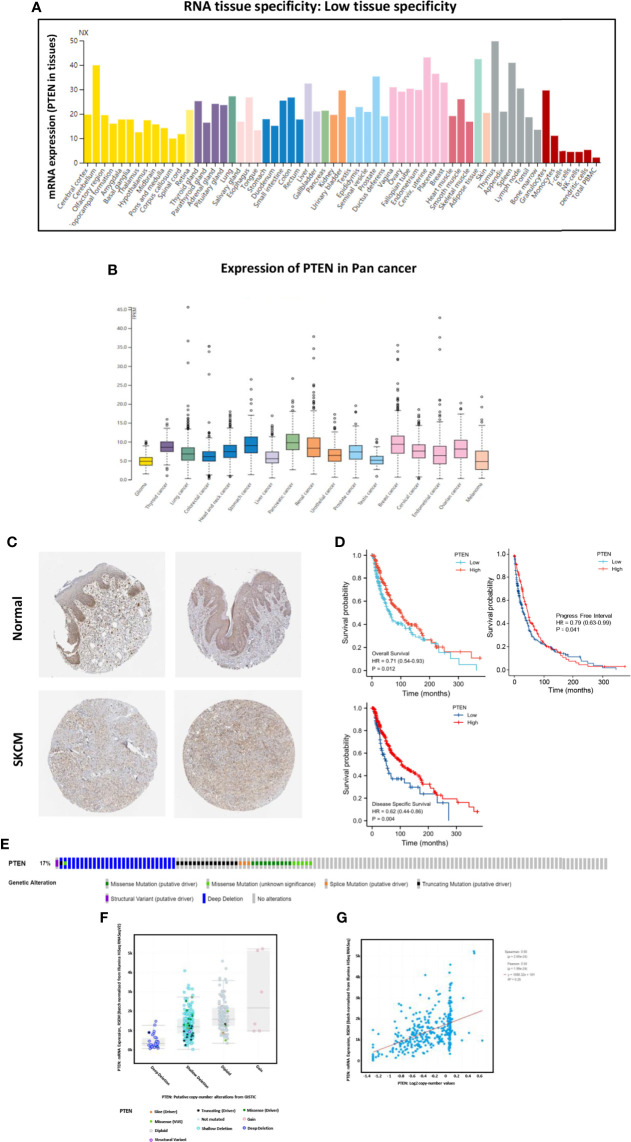
The tumor suppressor role of *PTEN* in SKCM. **(A)** Expression specificity of *PTEN* in various human tissues. **(B)** Expression distribution of *PTEN* in para-cancer patients. **(C)** Validation of the expression of *PTEN* at translational level using the Human Protein Atlas database (i.e., as determined by immunohistochemical staining). **(D)** Analysis of the relationship between the expression level of *PTEN* and patient survival rate, including overall survival, progress free interval, and disease specific survival. **(E)** Distribution of *PTEN* genomic alterations in SKCM according to The Cancer Genome Atlas (TGCA) database displayed as a cBioPortal OncoPrint plot. The relationship between *PTEN* copy number and mRNA expression are shown in the dot plot **(F)** and correlation plot **(G)**; they are all positively correlated.

We analyzed the expression of *PTEN* in both normal and cancer human tissues at the mRNA and protein levels using HPA database. The results showed that *PTEN* was expressed at a medium level in normal skin tissue (**Figure 2A**), but at low level in cutaneous melanoma (**Figure 2B**). Immunohistochemical staining results in the HPA database also confirmed a medium expression level of *PTEN* in normal tissues and low *PTEN* expression in SKCM tissues, consistent with the TCGA database results (**Figure 2C**, **Figure S1**, and **Table S1**). As *PTEN* is downregulated in SKCM, we analyzed the overall survival (OS) rate of SKCM patients with *PTEN* expression. The results suggested that low *PTEN* expression was associated with poor OS of SKCM patients (**Figure 2D**). Furthermore, we explored the possible mechanism of the low expression of *PTEN* in SKCM patients and analyzed the associated genome and copy number changes using cBioPortal. The OncoPrint plot showed the deletion of *PTEN* in the TCGA SKCM dataset (**Figure 2E**); moreover, as evidenced in **Figure 2F**, more than half of the SKCM dataset had *PTEN* deletions. Similarly, the mRNA expression levels in the *PTEN*-deficient SKCM dataset were significantly higher in the diploid and gain groups. Additionally, as the copy number of *PTEN* increased, the expression level of its mRNA also increased significantly (**Figure 2G**).

### Analysis of DElncRNAs, DEmiRNAs, and DEmRNAs

The above results strongly suggested that the *PTEN*-related ceRNA network could be used as a prognostic evaluation indicator for SKCM. Therefore, we analyzed cancer tissues and their adjacent tissues to determine whether they had differential *PTEN* expression. We first divided the TCGA data into *PTEN* ^high^ and *PTEN* ^low^ expression groups using adjusted p < 0.05 and |log FC| > 0.5 as screening conditions for DElncRNAs, DEmiRNAs, and DEmRNAs.

**Figure 3:**
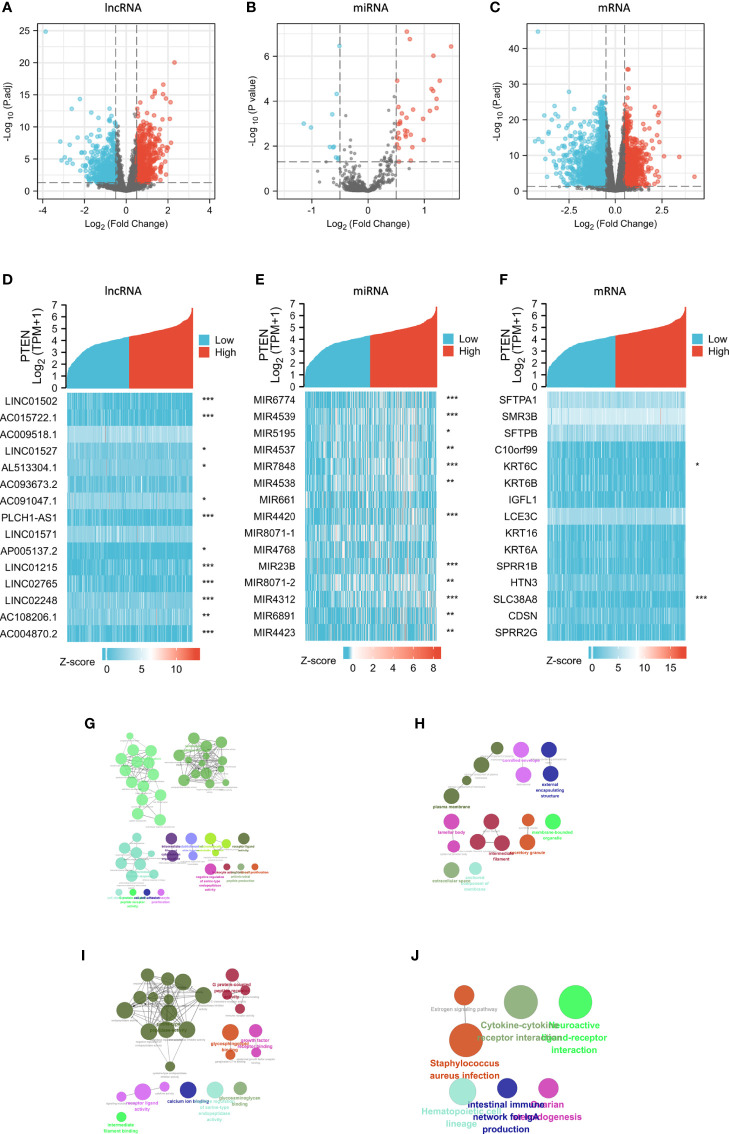
Analysis of DElncRNAs, DEmiRNAs, and DEmRNAs in groups characterized by *PTEN* ^high^ and *PTEN* ^low^ expression in the SKCM dataset. Red represents upregulated genes and blue indicates downregulated genes. Volcano plots describing **(A)** 1457 DElncRNAs (|log2 FC| > 0.5, adjusted p value < 0.05), **(B)** 44 DEmiRNAs (|log2 FC| > 0.5, adjusted p value < 0.05), and **(C)** 3339 DEmRNAs (|log2 FC| > 0.5, adjusted p value < 0.05). **(D–F)** The horizontal axis of the heatmap corresponds to groups and the vertical axis to 15 significant DEGs (lncRNAs, miRNAs, and mRNAs). **(G–J)** GO and KEGG analyses of significantly upregulated DEmRNAs in the *PTEN* ^low^ group using Cytoscape. The color indicates a class of biological functions, whereas the size of the circle indicates the degree of significance. *p < 0.05, **p < 0.01, ***p < 0.001.

There were 1457 DElncRNAs (779 upregulated and 678 downregulated), 44 DEmiRNAs (33 upregulated and 11 downregulated), and 3339 DEmRNAs (1129 upregulated and 2210 downregulated) in the *PTEN* ^high^ expression group (**Figures 3A-C**). A heatmap was then constructed for the top 15 lncRNAs, miRNAs, and mRNAs with significant differences in expression between the *PTEN* ^high^ and *PTEN* ^low^ groups (**Figures 3D-F**). Both the volcano map and heatmap were used to visualize the results obtained using the ggplot2 package of R.

We performed GO/KEGG analyses on selected DEGs using ClueGO plug-in of Cytoscape (25) (**Figures 3G-J**) and analyzed the different expression levels of lncRNAs, miRNAs, and mRNAs in SKCM and their adjacent tissues. In total, 584 DElncRNAs (226 upregulated and 358 downregulated), 23 DEmiRNAs (five upregulated and 18 downregulated), and 3102 DEmRNAs (1510 upregulated and 1592 downregulated) (**Figures S2A-C**).

### Construction of the lncRNA-miRNA-mRNA triple regulatory network

**Figure 4:**
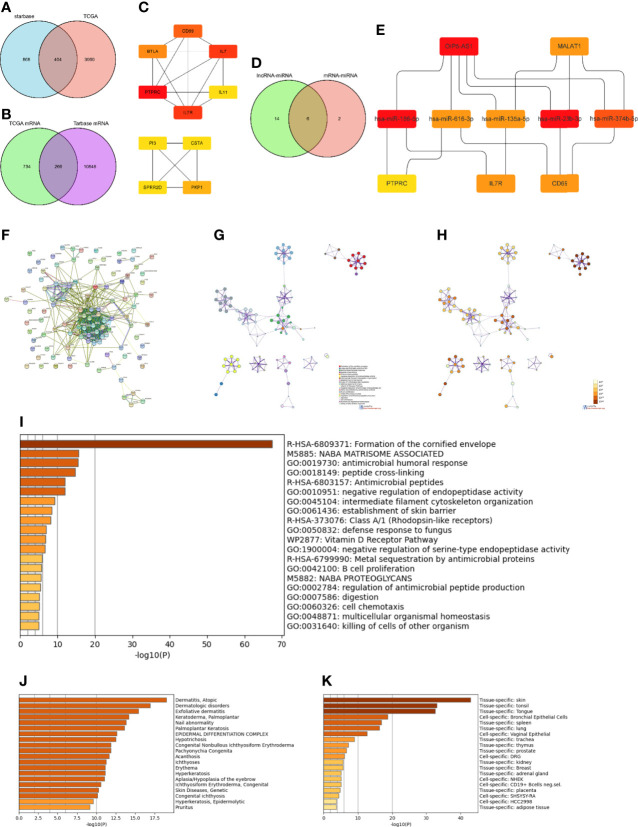
Construction and functional enrichment analysis of the lncRNA-miRNA-mRNA triple regulatory network. **(A)** Venndiagram of the 1272 lncRNAs predicted by the starBase database based on corresponding DEmiRNAs and 3464 DElncRNAs selected through the TCGA database analysis. **(B)** Venndiagram of 1000 DEmRNAs selected from TCGA and 11,114 mRNAs predicted from the DEmiRNAs in the TarBase database. **(C)** Interaction network analysis of core DEmRNAs obtained by Cytoscape. **(D)** Eight miRNAs predicted by the three most significant mRNAs intersected with the 20 miRNAs predicted from the top ten DElncRNAs. **(E)** Core ceRNA network predicted by the CytoHubba plug-in of Cytoscape. **(F)** Interaction regulatory network of 100 important DEGs related to the core ceRNA network obtained through STRING. **(G)** Enrichment analysis of the DEGs in **(F)** obtained by Metascape. **(H)** Significance of the enriched KEGG pathways. **(I)** Enriched terms in the DEmRNAs as obtained in Metascape, colored by p-values. **(J)** Enrichment analysis of DEmRNAs in DisGeNET, colored by p-values. **(K)** Enrichment analysis of DEmRNAs in PaGenBase, colored by p-values.

To construct the lncRNA-miRNA-mRNA regulatory network, 1272 lncRNAs were first predicted based on their corresponding DEmiRNAs. Next, 404 lncRNAs were obtained by intersecting the predicted lncRNAs with the 3464 DElncRNAs obtained from the TCGA database analysis (**Figure 4A**). The ten lncRNAs with the largest differences (*FTX*, *ENTPD1-AS1*, *AL365361.1*, *OIP5-AS1*, *SNAI3-AS1*, *AL132656.2*, *PSMD6-AS2*, *MALAT1*, *POLR2J4*, and *WDFY3-AS2*) were then further analyzed. Except for *AL132656.2*, all other lncRNAs have been reported to be associated with cancers (39–47). However, no studies have reported that these lncRNAs play a specific role in SKCM. Additionally, we predicted 11,114 mRNAs from the corresponding 1000 DEmiRNAs in the TCGA database and obtained 266 mRNAs by intersecting both datasets (**Figure 4B**). The top 50 DEmRNAs were then analyzed, and the three core mRNAs, i.e., those with the highest significance, were selected (*PTPRC*, *IL7R*, and *CD69*) (**Figure 4C**). We identified eight miRNAs corresponding to these three mRNAs (hsa-miR-186-5p, hsa-miR-616-3p, hsa-miR-23a-3p, hsa-miR-135a-5p, hsa-miR-339-5p, hsa-miR-4677-3p, hsa-miR-23b-3p, and hsa-miR-374b-5p). The top ten DElncRNAs were also used to predict 20 DEmiRNAs (**Figure 4D**), which were intersected with the miRNAs predicted by the mRNAs in the TCGA database to obtain six core miRNAs (**Figure 4D**). Overall, two lncRNAs (*OIP5-AS1* and *MALAT1*), five miRNAs (hsa-miR-186-5p, hsa-miR-616-3p, hsa-miR-135-5p, hsa-miR-23b-3p, and hsa-miR-374b-5p), and three mRNAs (*PTPRC*, *IL7R*, and *CD69*) were considered to form the ceRNA network (**Figure 4E**).

Using the STRING database (33), the interactions of 100 DEGs surrounding the final ceRNA network were analyzed (**Figure 4F**). Functional enrichment analysis of all related DEGs in the interaction network showed that these DEGs were mainly enriched in the organization of intermediate filaments in the cytoskeleton, establishment of the skin barrier, and B cell proliferation (**Figures 4G, H** and **Figures 4I-K**). Thus, these results indicate that the ceRNA network obtained is involved in immune response.

### Further analysis of DEGs in the ceRNA network and selection of an SKCM-specific prognostic model

**Figure 5:**
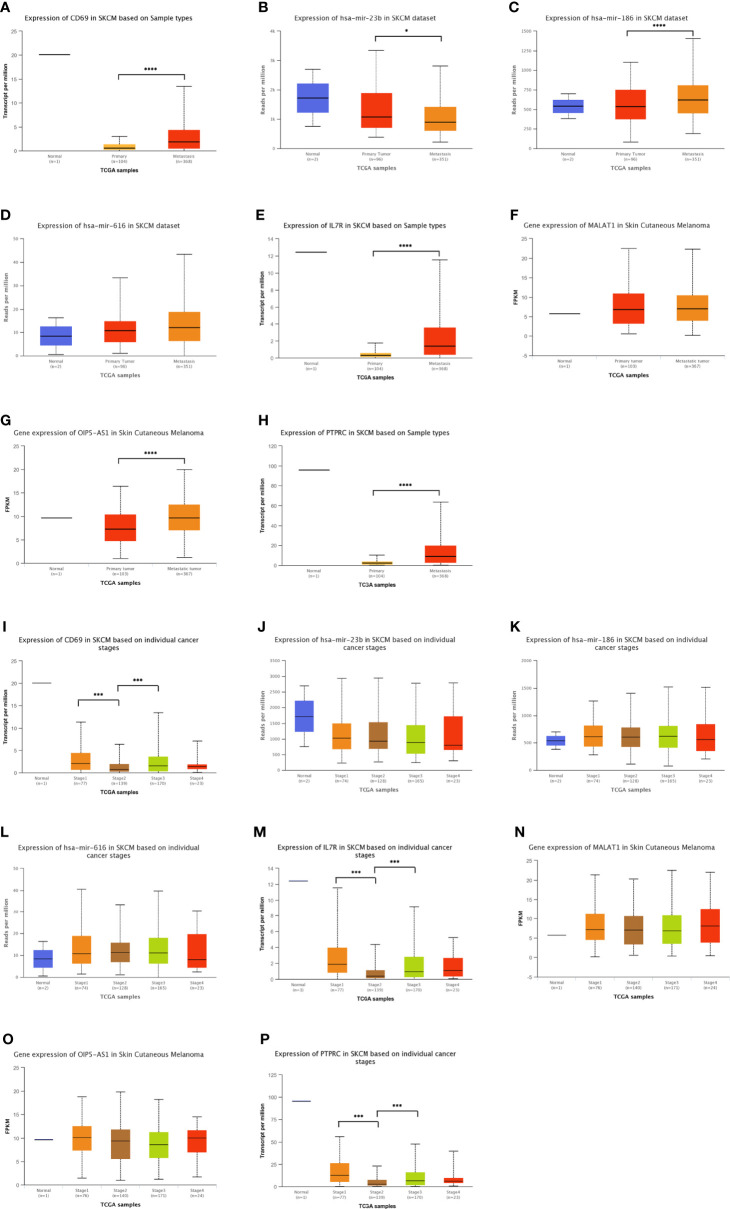
Distribution of the expression patterns of ten hub-genes in the triple regulatory network obtained from the UALCAN database. **(A–H)** The expression patterns of ten hub-genes in normal skin, primary SKCM, and metastatic SKCM datasets. **(I–P)** Expression patterns of the same hub-genes in tissue samples at different stages of SKCM. *p < 0.05, ***p < 0.001, ****p < 0.0001.

In addition to analyzing the expression levels of ceRNA-related genes in normal, primary SKCM, and metastasis groups (**Figures 5A-H**), we also analyzed the relationship between the different stages of SKCM and ceRNA-related genes (**Figures 5I-P**) to establish a ceRNA network with important prognostic value for SKCM. *OIP5-AS1*, hsa-mir-186, *IL7R*, *CD69*, and *PTPRC* were significantly upregulated, while hsa-mir-23b was significantly downregulated in the metastasis group compared with the primary SKCM group (**Figures 5A-H**). The expression levels of *CD69*, *IL7R*, and *PTPRC* decreased with the progression of tumor stages (**Figures 5I-P**). The expression levels of these genes were confirmed in the SKCM dataset of the TCGA and Genotype-Tissue Expression (GTEx) databases (https://gtexportal.org/home/; version 8) (48) (**Figure S2B**).

**Figure 6:**
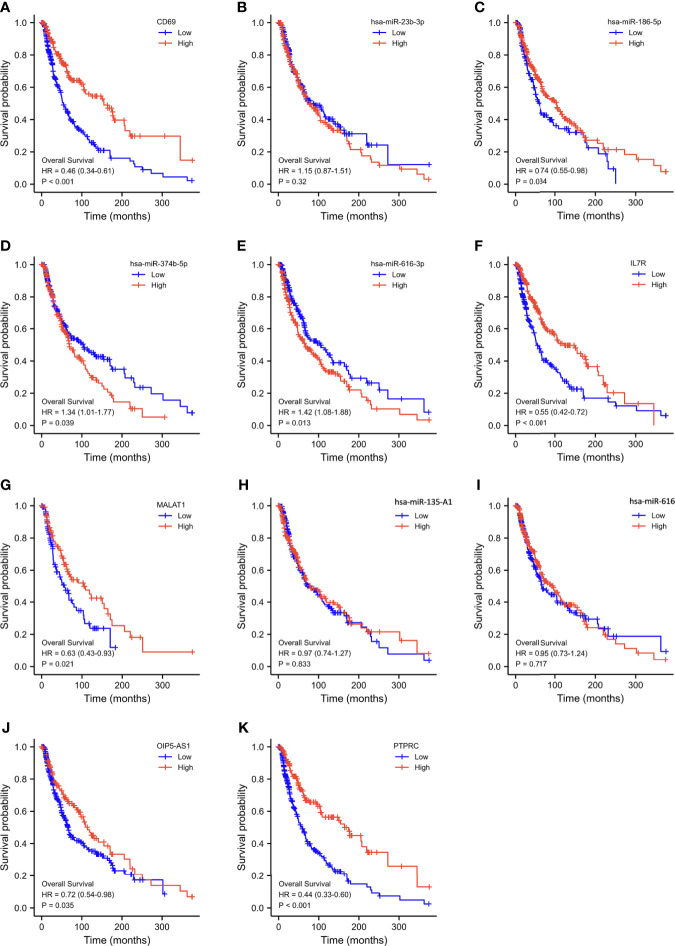
Overall survival analysis based on the expression levels of RNAs in the triple regulatory network. **(A–K)** Expression of ten hub-genes in the SKCM dataset with *CD69* ^high^ and *CD69* ^low^, hsa-miR-23b-3p^high^ and hsa-miR-23b-3p^low^, hsa-miR-186-5p^high^ and hsa-miR-186-5p^low^, hsa-miR-374b-5p^high^ and hsa-miR-374b-5p^low^, hsa-miR-616-3p^high^ and hsa-miR-616-3p^low^, *IL7R* ^high^ and *IL7R* ^low^, *MALAT1* ^high^ and *MALAT1* ^low^, hsa-miR-*135A1* ^high^ and hsa-miR-*135A1* ^low^, hsa-miR-*616* ^high^ and hsa-miR-*616* ^low^, *OIP5-AS1* ^high^ and *OIP5-AS1* ^low^, and *PTPRC* ^high^ and *PTPRC* ^low^ expression groups, which were compared using a Kaplan–Meier survival curve for TCGA SKCM patient cohort. The horizontal axis indicates the overall survival time in months and the vertical axis represents the survival rate.

Using Kaplan–Meier analysis and log-rank test, the expression levels of these DEGs were correlated with the OS rate of SKCM patients (**Figures 6A-K**). Except for hsa-miR-23b-3p, hsa-miR-135a-5p, and hsa-miR-616, the other RNAs involved in the ceRNA network significantly affected the OS rate of SKCM patients. Low expression of *CD69*, hsa-miR-186-5p, *IL7R*, *MALAT1*, *OIP5-AS1*, and *PTPRC* and high expression of hsa-miR-374b-5p and hsa-miR-616-3p were associated with poor OS of SKCM patients.

**Figure 7:**
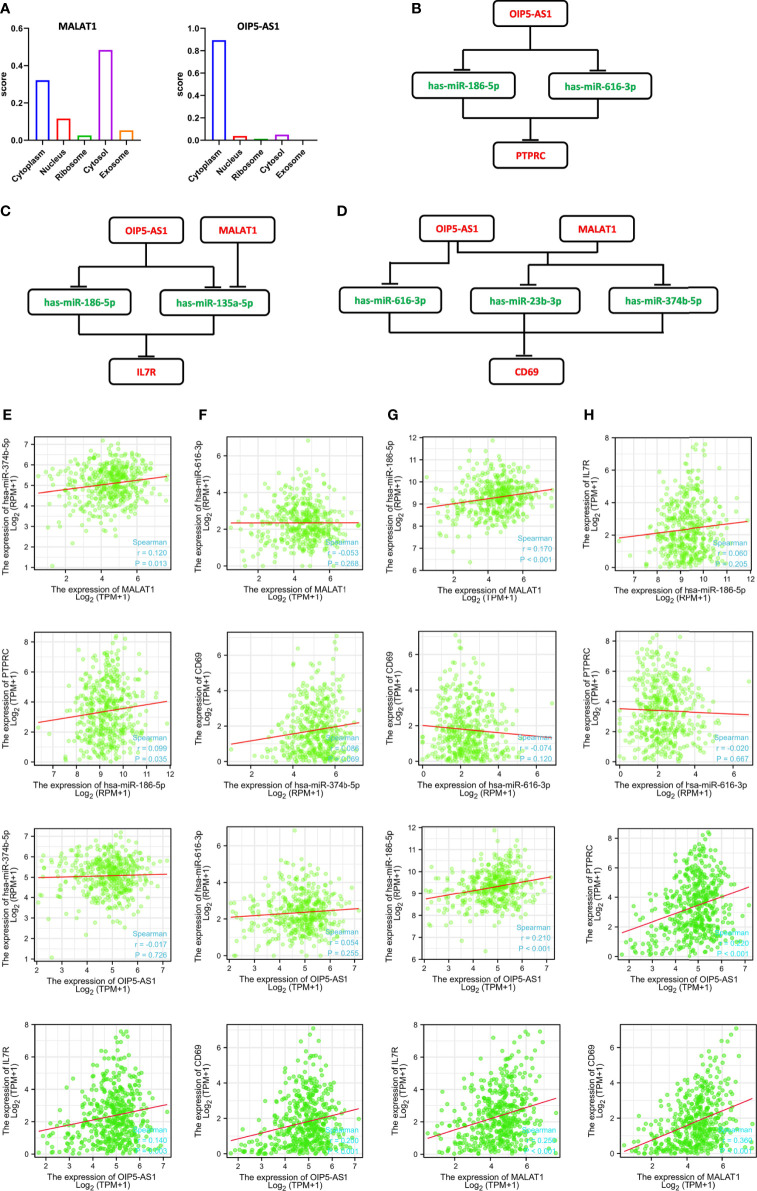
Construction and correlation analysis of the ceRNA network. **(A)** Cellular localization for two hub-lncRNAs (*MALAT1* and *OIP5-AS1*) predicted using lncLocator. **(B–D)** Schematic representation of the ceRNA networks. Green indicates downregulated and red indicates upregulated RNAs in SKCM. **(E–H)** Interaction correlation analysis between the ten predicted RNAs in SKCM, consistent with the predicted ceRNA networks.

The functions of lncRNAs are determined by their location in the cell. We analyzed the cellular localization of *MALAT1* and *OIP5-AS1*. *MALAT1* was mainly expressed in the cytoplasm and cytosol, whereas *OIP5-AS1* was mainly expressed in the cytoplasm (**Figure 7A**), as predicted using lncLocator. These results indicated that these two lncRNAs can affect the binding of miRNAs through sponge adsorption, thereby affecting the expression of *CD69*, *IL7R*, and *PTPRC*. Therefore, we constructed a ceRNA network for *MALAT1* and *OIP5-AS1* (**Figures 7B–D**). Through correlation analysis, we found that *OIP5-AS1* was positively correlated with *CD69*, *IL7R*, and *PTPRC* and *MALAT1* was positively correlated with *CD69* and *IL7R* (**Figures 7E–H**). These three ceRNA axes could serve as potential prognostic prediction models. Additionally, we also analyzed the relationship between the expression levels in the ceRNA network and the different expression levels of *PTEN*, which demonstrated that the expression of *CD69*, *IL7R*, *MALAT1*, hsa-miR-186-5p, *OIP5-AS1*, and *PTPRC* was positively correlated with *PTEN* expression (**Figure S3**). Furthermore, we analyzed the expression levels of DElncRNAs (*OIP5-AS1* and *MALAT1*) and DEmRNAs (PTPRC, IL7R, and CD69) in SKCM and normal skin samples in the TCGA and GTEx databases, respectively. The results showed that *OIP5-AS1* and *MALAT1* were both downregulated in SKCM samples, and *PTPRC* and *IL7R* also showed low expression. In addition, using the GEO dataset, we verified that the expression levels of *PTPRC*, *IL7R*, and *CD69* DEmRNAs were downregulated in SKCM (**Figures S4A, B**).

### Clinical significance of the three ceRNAs axes in SKCM

To confirm whether the three ceRNA axes are affected by clinical factors in patients with SKCM, we analyzed the correlation between the clinical factors and related RNAs in the three ceRNA axes. *OIP5-AS1* expression was correlated with the T stage (p = 0.026), N stage (p = 0.011), pathologic stage (p = 0.029), and Breslow depth (p =0.006) of SKCM (**Table S2**). However, there was no significant correlation between the expression levels of *MALAT1* and these clinical factors (**Table S3**). *CD69* expression was significantly correlated with the T stage (p < 0.001) and Breslow depth (p < 0.001) (**Table S4**). T stage (p < 0.001), age (p = 0.019), and Breslow depth (p < 0.001) had a significant correlation with *IL7R* expression (**Table S5**). T stage (p < 0.001), age (p = 0.011), and Breslow depth (p < 0.001) were also significantly correlated with *PTPRC* expression (**Table S6**).

Univariate and multivariate Cox analyses were used to determine the relationships between clinically relevant features and prognosis of SKCM. Results from *MALAT1*, *OIP5-AS1*, *IL7R*, *CD69*, and *PTPRC* single-factor Cox model analysis and *MALAT1*, *IL7R*, *CD69*, and *PTPRC* multi-factor Cox model analysis showed that the TNM staging of cancer patients in the TCGA cohort, age, pathologic stage, and Breslow depth were closely related to the OS rate (p < 0.05) (**Table S7-12**). The expression level of *MALAT1* (hazard ratio = 0.840, p = 0.005), *PTPRC* (hazard ratio = 0.817, p < 0.001), *IL7R* (hazard ratio = 0.841, p < 0.001), and *CD69* (hazard ratio = 0.747, p < 0.001) was significantly negatively correlated with the OS rate. However, the expression level of *OIP5-AS1* (hazard ratio = 0.850, p = 0.059) was not significantly correlated with the OS rate. The results were obtained using the multivariate Cox model of these genes. The expression levels of *IL7R*, *CD69*, and *PTPRC* and the TNM stages of the tumor were significantly related to the OS rate of SKCM patients. Therefore, we hypothesize that *IL7R*, *CD69*, and *PTPRC* can become important indicators for SKCM prognosis.

### Verification of abnormally high expression of *IL7R*, *CD69*, and *PTPRC*

We conducted a detailed analysis of *IL7R*, *CD69*, and *PTPRC* to explore the mechanism underlying the ceRNA network in SKCM. Through analysis of the data in the CCLE database, we found that *IL7R*, *CD69*, and *PTPRC* were highly expressed in SKCM cell lines (**Figure S5**), supporting our TCGA analysis (**Figure 5**).

Abnormal gene expression may be caused by gene mutations. The OncoPrint plot showed the amplification of *IL7R*, *CD69*, and *PTPRC* in SKCM in the TCGA dataset (**Figure S6A**). In the SKCM dataset there was no significant correlation between the expression and copy number of these genes (**Figure S6B**).

### *IL7R*, *CD69*, and *PTPRC* methylation and expression levels

**Figure 8:**
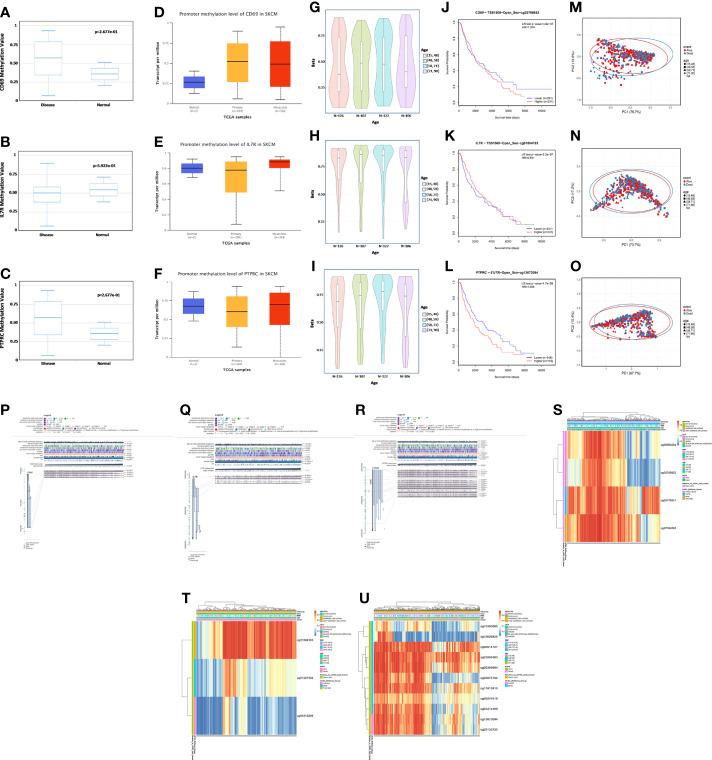
Methylation analysis of *CD69*, *IL7R*, and *PTPRC.* **(A–C)** Methylation levels of *CD69*, *IL7R*, and *PTPRC* in normal and disease datasets assessed using DiseaseMeth (version 2.0). **(D–F)** Promoter methylation level of *CD69*, *IL7R*, and *PTPRC* in normal skin, primary SKCM, and metastasis datasets evaluated using UALCAN. **(G–I)** Methylation level of *CD69*, *IL7R*, and *PTPRC* in normal tissues of people with different ages. **(J–L)** Correlation between *CD69*, *IL7R*, and *PTPRC* methylation levels and the survival curve of SKCM patients. **(M–O)** In the three genes, differences between the proportion of PC1 and PC2 is high, showing that the level of methylation has a significant association with survival outcomes of SKCM patients. **(P–R)** Methylation sites of CD69, IL7R, and PTPRC and their expression levels as visualized using MEXPRESS. The expression of *CD69*, *IL7R*, and *PTPRC* is illustrated by the blue line in the center of the plot. Pearson’s correlation coefficients and p-values for methylation sites and query gene expression are shown on the right side. **(S–U)** Heatmap of the expression of different methylation sites in *CD69*, *IL7R*, and *PTPRC* in different samples; the ethnicity, race, body mass index, age, event, and other variables are indicated, and the whole analysis was carried using the MethSurv database.

Exploring the correlation between the expression and methylation levels of *IL7R*, *CD69*, and *PTPRC* may help revealing the possible mechanism for the abnormal upregulation of these genes in SKCM patients. Therefore, we analyzed the methylation levels of these three genes in normal and SKCM tissues and their correlation with age (**Figures 8A-I**). There was no significant difference in the methylation levels of these genes in normal and SKCM tissues (neither primary nor metastatic tumor tissues) (**Figures 8A-F**). Moreover, there was no difference in the methylation levels of these three genes with age (**Figures 8G-I**). However, there was a significant negative correlation between the methylation levels of the three genes and the OS rate of SKCM patients—higher methylation levels were associated with lower OS rate (**Figures 8J-L**). The methylation levels also had a significant impact on OS outcomes (**Figures 8M-O**). We found four methylation sites in *CD69* (cg05590294, cg25769852, cg05179921, and cg07354583); cg05590294 was positively correlated with the expression level of *CD69*, and the remaining three sites were negatively correlated with its expression level. Three methylation sites (cg01804183, cg01027405, and cg04312209) were observed in *IL7R*; cg04312209 was negatively correlated with the expression level of *IL7R* while cg01804183 and cg01027405 were positively correlated. We observed 11 methylation sites in *PTPRC*; cg12390585, cg15626828, and cg22073152 were negatively correlated with the expression level of *PTPRC*, while the remaining sites were positively correlated (**Figures 8P-U**).

### Expression levels of *IL7R*, *CD69*, and *PTPRC* in SKCM and immune infiltration

**Figure 9:**
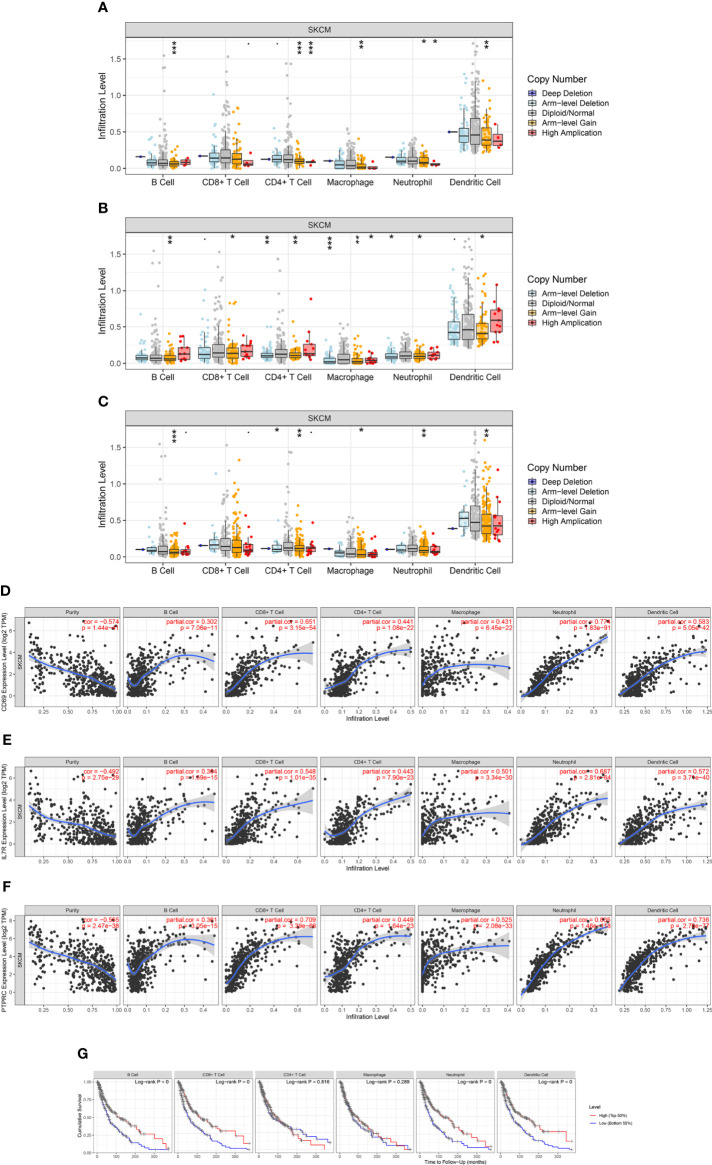
Correlation analysis of *CD69*, *IL7R*, and *PTPRC* expression and immune infiltration in SKCM. **(A–C)** Relationship between *CD69*, *IL7R*, and *PTPRC* gene copy number and immune cell infiltration levels in SKCM cohorts, including B cells, CD4+ T cells, macrophages, neutrophils, and dendritic cells. **(D–F)** Interaction correlation of *CD69*, *IL7R*, and *PTPRC* expression with immune infiltration level in SKCM, including B cells, CD4+ T cells, macrophages, neutrophils, and dendritic cells. **(G)** Kaplan–Meier plots showing the impact of immune infiltration level of various cells, including B cells, CD4+ T cells, macrophages, neutrophils, and dendritic cells, on the overall survival rate of SKCM patients. *p < 0.05, **p < 0.01, ***p < 0.001.

To evaluate the relationship between the expression levels of *IL7R*, *CD69*, and *PTPRC* and the level of SKCM immune infiltration, we performed a correlation analysis using the TIMER database. The results of the somatic copy number alterations module analysis showed that the level of immune cell infiltration in SKCM was related to the number of *CD69* gene copies and positively correlated with the copy numbers of *IL7R* and *PTPRC* genes in B cells, CD4+ T cells, macrophages, neutrophils, and dendritic cells (**Figures 9A-F**). The results also indicated that low levels of immune cell infiltration of B cells, CD8+ T cells, neutrophils, and dendritic cells were associated with poor prognosis in SKCM patients with an OS period of two years or less (p < 0.05) (**Figure 9G**). Our results indicated that the predicted ceRNA network is closely related to tumor immune infiltration, and the positive correlation between the two implies that the ceRNA network may affect the prognosis of SKCM by regulating the level of tumor-infiltrating immune cells. However, this causal relationship requires further exploration and experimental verification.

### Enrichment analysis of the related functions of *OIP-AS1*, *MALAT1*, *IL7R*, *CD69*, and *PTPRC* in SKCM

We used GO and KEGG enrichment analyses to explore the putative functions of *OIP-AS1*, *MALAT1*, *IL7R*, *CD69*, and *PTPRC* genes in SKCM (**Figure S7**). GO enrichment analysis included biological process (BP), cell component (CC), and molecular function (MF) terms. For *OIP-*AS1, enriched BP terms included “epidermis development”, “skin development”, and “epidermal cell differentiation”. CC terms included “lamellar body”, “anchored component of membrane”, and “basolateral plasma membrane” while MF terms included “structural constituent of epidermis”, “peptidase regulator activity”, and “receptor ligand activity”. KEGG enriched pathways mainly included “alpha-linolenic acid metabolism”, “arachidonic acid metabolism”, and “carbohydrate digestion and absorption”. The GO enrichment analysis of *MALAT1* revealed “epidermis development”, “skin development”, and “epidermal cell differentiation” as the main BP terms, “chylomicron”, “GABA-A receptor complex”, and “GABA receptor complex” as the main CC terms, and “inhibitory extracellular ligand-gated ion channel activity”, “GABA-A receptor activity”, and “chloride channel activity” as the main MF terms. KEGG enrichment mainly occurred on pathways such as “primary immunodeficiency”, “nicotine addiction”, and “cholesterol metabolism”. The BP enriched terms of *IL7R* were “regulation of lymphocyte activation”, “leukocyte migration”, and “immune response-activating cell surface receptor signaling pathway”. CC enriched terms were mainly “external side of plasma membrane”, “plasma membrane receptor complex”, and “immunoglobulin complex” and MF terms were mainly enriched in “regulation of lymphocyte activation”, “immune response-activating cell surface receptor signaling pathway”, and “positive regulation of cell activation”. KEGG enriched pathways were mainly “external side of plasma membrane”, “plasma membrane receptor complex”, and “immunoglobulin complex”. The top BP terms enriched for *PTPRC* were “regulation of lymphocyte activation”, “immune response-activating cell surface receptor signaling pathway”, and “positive regulation of cell activation”. The top CC terms enriched were “external side of plasma membrane”, “plasma membrane receptor complex”, and “immunoglobulin complex” and MF enriched terms were “antigen binding”, “receptor ligand activity”, and “cytokine receptor”. The main KEGG enriched pathways were “cytokine-cytokine receptor interaction”, “hematopoietic cell lineage”, and “cell adhesion molecules”. The enriched BP terms of *CD69* were mainly “regulation of lymphocyte activation”, “immune response-activating cell surface receptor signaling pathway”, and “lymphocyte-mediated immunity”. The main enriched CC terms were “external side of plasma membrane”, “immunoglobulin complex”, and “plasma membrane receptor complex” and the main MF terms enriched were “antigen binding”, “cytokine receptor binding”, and “carbohydrate binding”. KEGG enriched pathways were mainly “cytokine-cytokine receptor interaction”, “chemokine signaling pathway”, and “cell adhesion molecules”.

## Discussion

Melanoma is one of the most fatal skin cancers. It has a low incidence but a high degree of malignancy, early metastasis, and high mortality. Most melanomas originate in the skin, are more common in men than in women, and frequently affect the feet and lower limbs, followed by the trunk, head and neck, and upper limbs. SKCM may also originate in the eyes and nasal cavity and has a high metastasis rate (3). Current clinical treatments for SKCM are mainly surgical resection, targeted therapy, and immunotherapy. However, problems such as metastasis and drug resistance are prone to occur, which affect the therapeutic efficacy and prognosis of patients (49). In-depth exploration of the pathogenesis of SKCM and development of new therapeutic and prognostic targets are therefore essential. With the continuous improvement in genomics technology, new types and functions of non-coding RNAs are constantly being identified. Among them, lncRNAs, a large category of non-coding RNAs, are abundantly expressed in various tissues of the human body. LncRNAs mainly function through a ceRNA mechanism. LncRNAs distributed in the cytoplasm can bind with miRNAs to affect the expression of target genes and biological functions (12). In tumors, lncRNAs can affect tumor cell growth, migration, and invasion through the ceRNA mechanism (50, 51). As a “star” tumor suppressor gene, *PTEN* is generally reported to be downregulated in tumor tissues, leading to the malignant proliferation of tumor cells (52, 53). Research on the ceRNA network related to *PTEN* in SKCM may not only reveal more complex tumor pathological processes but also lead to the development of novel clinical diagnoses, treatments, and prognostic indicators.

Using Cytoscape, we obtained a ceRNA network composed of 20 lncRNAs, six miRNAs, and eight mRNAs. Enrichment analyses revealed that these regulatory networks are mainly related to “establishment of skin barrier”, “multicellular organismal homeostasis”, “G protein-coupled peptide receptor activity”, and “growth factor receptor binding”. Furthermore, we analyzed the key regulatory network using hub analysis to obtain a core ceRNA regulatory network composed of two lncRNAs, five miRNAs, and three mRNAs. Subsequently, we analyzed the cellular localization of *MALAT1* and *OIP5-AS1*. As the ceRNA mechanism primarily occurs in the cytoplasm, the two lncRNAs were mainly distributed in the cytoplasm or cytosol. Thus, we obtained a ceRNA network composed of *OIP5-AS1-PTPRC*/*IL7R*/*CD69* and *MALAT1-IL7R*/*CD69*, which affects SKCM prognosis.

Several key molecules in the ceRNA network, such as *OIP5-AS1* and *MALAT1*, have been reported to be related to tumor pathology. High expression of *OIP5-AS1* promotes the growth and migration of melanoma cells (54). *MALAT1* promotes the growth and migration of melanoma cells by interacting with miRNAs (55). *PTPRC* is used as a prognostic indicator of cervical cancer (56), but its specific role in SKCM is still unclear. *CD69* is significantly related to the tumor immune microenvironment and participates in the tumor immune infiltration process in melanoma (57). *OIP5-AS1* protects microglia from hypoxia/ischemia-induced neuronal damage by sponging miR-186-5p (58). It also affects the pathological progression of atherosclerosis by acting as a miR-135a-5p sponge (59). Similarly, silenced *MALAT1* reduced myocardial ischemia-reperfusion injury in rat cardiomyocytes by regulating the miR-135a-5p/HIF1AN axis (60). Although there is no report on the ceRNA axis being directly related to *OIP5-AS1* or *MALAT1*, both hsa-miR-23b-3p and hsa-miR-374b-5p are involved in tumor progression (61, 62).

Previous research has reported that the abnormal expression of certain genes in tumors is unrelated to changes in gene copy number. Rather, it is regulated by epigenetics. The expression of the three significant DEmRNAs found here was not significantly associated with gene copy number, and their expressions were regulated by related lncRNAs and miRNAs. Epigenetic modification is a recently proposed mechanism of gene regulation. In addition to the regulation of non-coding RNAs, most studies on epigenetics have focused on DNA methylation (63, 64). Changes in methylation levels are common in tumors and affect tumor outcomes (65). In the present study, we explored the effect of DNA methylation on the expression of the genes related to the non-coding RNAs but found no significant methylation of *CD69*, *IL7R*, and *PTPRC* in tumor tissues compared with that of normal tissues. This may be related to the volume of the included datasets. Nevertheless, methylation levels were significantly negatively correlated with the OS prognosis of SKCM. Therefore, we analyzed the methylation sites as this information may be used as basis to customize targeted therapy for other tumors and for further tumor-related methylation research in the future.

SKCM is one of the most immunogenic tumors and therefore highly likely to respond to immunotherapy. We analyzed the correlation between related target genes and immune infiltration and found that the expression levels of *CD69*, *IL7R*, and *PTPRC* were all related to the level of immune cell infiltration. Further analysis revealed that these three genes were positively correlated with the infiltration levels of B cells, CD8+ T cells, CD4+ T cells, macrophages, neutrophils, and dendritic cells. These immune-infiltrating cells were significantly related to the OS rate and prognosis of tumor patients. This indicates that the *OIP5-AS1-PTPRC*/*IL7R*/*CD69* and *MALAT1-IL7R*/*CD69* axes in SKCM may affect the tumor immune microenvironment, leading to SKCM progression.

We further performed GO and KEGG enrichment analyses on the core lncRNAs and mRNAs and found that *OIP-AS1* KEGG was mainly enriched in the IL-17 signaling pathway and *MALAT1* in the primary immunodeficiency pathway. GO (BP, CC, and MF) analysis demonstrated that *OIP-AS1* was mainly enriched in epidermal cell differentiation, cell-cell junction, and peptidase regulator activity. *MALTA1* was enriched in humoral immune response, collagen-containing extracellular matrix, and anion transmembrane transporter activity. KEGG analysis showed that *IL7R*, *PTPRC*, and *CD69* were mainly enriched in chemokine signaling and cell adhesion pathways. GO analysis showed that the three genes were mainly enriched in immune-related terms such as lymphocyte-mediated immunity, immunoglobulin complex, and immunoglobulin receptor binding. These results suggest that these genes are related to the maintenance of the normal structure and function of the skin and immune response.

The *OIP5-AS1-PTPRC*/*IL7R*/*CD69* and *MALAT1-IL7R*/*CD69* axes obtained here provide possible clinical prognostic indicators for SKCM. However, we could not perform any further clinical experimental verification. Moreover, the specific biological and molecular functions of the *OIP5-AS1-PTPRC*/*IL7R*/*CD69* and *MALAT1-IL7R*/*CD69* axes in SKCM require further investigation.

In conclusion, we analyzed the *PTEN*-related ceRNA network in SKCM through a variety of bioinformatics analysis methods and obtained the specific expression patterns of the axes *OIP5-AS1-PTPRC*/*IL7R*/*CD69* and *MALAT1-IL7R*/*CD69* in SKCM. These results provide potential indicators for the clinical prognosis of SKCM and lays theoretical foundation for exploring the pathogenesis of SKCM.

## Data availability statement

The original contributions presented in the study are included in the article/**Supplementary Material**. Further inquiries can be directed to the corresponding author.

## Author contributions

XZ, study design, conceptualization, conducting the study, data collection and analysis, methodology, and writing the original draft of the manuscript. RR, conducting the study, data collection and analysis, and writing the original draft of the manuscript. SX, conducting the study and writing, reviewing, and editing the manuscript. WS, conducting the study, writing, reviewing and editing the manuscript, and data analysis. DJ, visualization and writing, reviewing, and editing the manuscript. XX, study design, conceptualization, and writing, reviewing, and editing the manuscript. All authors have contributed to the manuscript and approved the submitted version.

## Funding

This work was supported by grants from the Hunan Natural Science Foundation (2021JJ31133, 2019JJ40474), The science and technology innovation Program of Hunan Province (2021RC2024), Project funded by China Postdoctoral Science Foundation (2021M703644), National key research and development program of China (2020YFC2008205), National Natural Science Foundation of China (81974134, 81400442, 82171058), and Key R&D plan of Hunan Province of China (2020SK2076).

## Conflict of interest

The authors declare that the research was conducted in the absence of any commercial or financial relationships that could be construed as a potential conflict of interest.

## Publisher’s note

All claims expressed in this article are solely those of the authors and do not necessarily represent those of their affiliated organizations, or those of the publisher, the editors and the reviewers. Any product that may be evaluated in this article, or claim that may be made by its manufacturer, is not guaranteed or endorsed by the publisher.

## Abbreviations

BP, biological process; CC, cell component; CCLE, Cancer Cell Line Encyclopedia; ceRNAs, competing endogenous RNAs; DE, differentially expressed; DEGs, differentially expressed genes; DElncRNAs, differentially expressed long non-coding RNAs; DEmRNAs, differentially expressed mRNAs; DEmiRNAs, differentially expressed microRNAs; FC, fold-change; GEO, Gene Expression Omnibus; GO, gene ontology; GTEx, Genotype-Tissue expression database; HPA, Human Protein Atlas; KEGG, Kyoto Encyclopedia of Genes and Genomes; lncRNAs, long non-coding RNAs; MF, molecular function; miRNAs, microRNAs; OS, overall survival; PTEN, phosphatase and tensin homolog; RNA-Seq, RNA sequencing; SKCM, skin cutaneous melanoma; TCGA, The Cancer Genome Atlas; TIMER, Tumor Immune Estimation Resource; UALCAN, The University of Alabama at Birmingham Cancer data analysis portal.
