# Supplementary material for: Integrated analysis to reveal potential therapeutic targets and prognostic biomarkers of skin cutaneous melanoma
Source: Front Immunol. 2022 Aug 11;13:914108. doi: 10.3389/fimmu.2022.914108 (PMC9402985; doi:10.3389/fimmu.2022.914108)
Supplement: Supplementary file 1 [file DataSheet_1.docx]

| Figure 1 | Flowchart of construction and analysis of the ceRNA network in SKCM |  |
| --- | --- | --- |
| Figure 2 | The expression and role of PTEN in SKCM |  |
| Figure 3 | Analysis of DElncRNAs, DEmiRNAs and DEmRNAs, between the expression of PTEN^high^ and PTEN^low^ in the SKCM dataset |  |
| Figure 4 | Construction and functional enrichment analysis of the lncRNA-miRNA-mRNA triple regulatory network |  |
| Figure 5 | The distribution of 10 hub-RNA expression patterns from the triple regulatory network in UALCAN database |  |
| Figure 6 | Overall survival analysis for the RNAs in the hub triple regulatory network |  |
| Figure 7 | Construction and correlation analysis of the ceRNA network |  |
| Figure 8 | Methylation analysis of CD69, IL7R, and PTPRC |  |
| Figure 9 | Correlation analysis of CD69, IL7R, and PTPRC expression and immune infiltration in SKCM |  |
| Table 1 | The expression of PTEN in normal samples and SKCM samples | referred here as Table S1 |
| Table 2 | Logistics regression of OIP5-AS1 single gene | referred here as Table S2 |
| Table 3 | Logistics regression of MALAT1 single gene | referred here as Table S3 |
| Table 4 | Logistics regression of CD69 single gene | referred here as Table S4 |
| Table 5 | Logistics regression of IL7R single gene | referred here as Table S5 |
| Table 6 | Logistics regression of PTPRC single gene | referred here as Table S6 |
| Table 7 | The results of PTEN, IL7R, PTPRC, CD69, MALAT1 and OIP5-AS1 with multiple factors in SKCM (Cox regression model) | referred here as Table S7 |
| Table 8 | Single factor and multiple factors of MALAT1 in SKCM (Cox regression model) | referred here as Table S8 |
| Table 9 | Single factor and multiple factors of OIP5-AS1 in SKCM (Cox regression model) | referred here as Table S9 |
| Table 10 | Single factor and multiple factors of PTPRC in SKCM (Cox regression model) | referred here as Table S10 |
| Table 11 | Single factor and multiple factors of IL7R in SKCM (Cox regression model) | referred here as Table S11 |
| Table 12 | Single factor and multiple factors of CD69 in SKCM (Cox regression model) | referred here as Table S12 |
| Figure S1 | The expression of PTEN in normal and tumor tissues separately, analyzed by TCGA and GTEx databases |  |
| Figure S2 | Volcano plots of DElncRNAs, DEmiRNAs, and DEmRNAs between SKCM samples and adjacent non-tumor samples |  |
| Figure S3 | Expression profile of ten hub genes in samples with different PTEN expression levels |  |
| Figure S4 | The expression value of hub DElncRNAs (OIP5-AS1 and MALAT1), DEmRNAs (PTPRC, IL7R and CD69) in SKCM and non-tumor samples respectively. (B) The expression value of DEmRNAs (PTPRC, IL7R and CD69) in SKCM and non-tumor samples of GEO dataset |  |
| Figure S5 | The expression levels of three differential mRNAs (IL7R, CD69 and PTPRC) in different cancer cell lines of human organs |  |
| Figure S6 | The mutation status of CD69, IL7R and PTPRC in SKCM |  |
| Figure S7 | Functional enrichment analysis of 2 DElncRNAs and 3 DEmRNAs |  |
