# Supplementary material for: Integrated analysis to reveal potential therapeutic targets and prognostic biomarkers of skin cutaneous melanoma
Source: Front Immunol. 2022 Aug 11;13:914108. doi: 10.3389/fimmu.2022.914108 (PMC9402985; doi:10.3389/fimmu.2022.914108)
Supplement: Supplementary file 2 [file DataSheet_2.docx]

**Methods**

1. **HPA database analysis**

Firstly, “PTEN” was entered in the “SEARCH” box on the official HPA website (httpa://www.proteinatlas.org, version 21.0) (1). “PTEN” was selected as “GENE^i^” and “Phosphatase and tensin homolog” was selected as “Gene description^i^” in the “GENES FOUND^!^” search results. Secondly, an “RNA EXPRESSION OVERVIEW^i^” graph of PTEN in tissues can be obtained by selecting the “TISSUE^i^” option (Fig. 2A). Then “SKIN” was selected from the drop-down menu of the “TISSUES” option on the left, and the normal skin tissue immunohistochemical figures can be obtained in the “SKIN 2 - Antibody staining^i^” box (Fig. 2C normal group). By clicking on the selected immunohistochemical picture, detailed information such as staining antibody, gender, age, and Patient ID can be obtained. The RNA expression of PTEN in 17 tumors in “TCGA data^i^” can be obtained by selecting the “PATHOLOGY” option (Fig. 2B). Then by selecting the “SKIN CANCER” option in the “CANCER” drop-down menu on the left, the immunohistochemical staining pictures of SKCM tumor tissue obtained in the “SKIN CANCER – Protein expression^i^” section (Fig. 2C SKCM). By clicking on the selected tumor immunohistochemistry image, detailed information such as staining antibody, gender, age and Patient ID can be obtained (Table. S1).

1. **TCGA survival analysis**

The FPKM data from high-throughput (HT) RNA-Seq were downloaded from the TCGA (https://portal.gdc.cancer.gov/, version 1.28.0) SKCM project, then it was converted into TPM data, and performed log2 transformation. After the processed data were imported into R (version 3.6.3), the survival data were statistically analyzed through the survival package (version 3.2.10) (2). The selected prognosis types were Overall Survival, Disease Specific Survival and Progress Free Interval respectively, and the statistical results were visualized by the survminer package (version 0.4.9) (3) (Fig 2D). In the Overall Survival analysis of SKCM, the median survival time of the PTEN^low^ group was 47.6 (34.4-67.6); the median survival time of the PTEN^high^ group was 96.3 (70-114.1). Information on follow-up time or prognostic outcomes was missing in 15 samples. The results of Log-rank test showed that the difference of survival time distribution of two groups was statistically significant (P=0.005); the Cox regression results showed that the difference of survival time distribution of two groups was statistically significant (P=0.006). The results suggested that the prognosis of PTEN^high^ group was better. In the Disease Specific Survival analysis of SKCM, the median survival time of the PTEN^low^ group was 50.8 (36.8-135.4). The median survival time in the PTEN^high^ group was 104.7 (82.3-154.9). The information of 21 samples on follow-up time or prognostic outcomes was missing. The result of the Log-rank test indicated that the difference in the distribution of survival time in the two groups was statistically significant (P = 0.004); the Cox regression result indicated that the difference in the distribution of the survival time in the two groups was statistically significant (P = 0.004). The results suggested that the prognosis of PTEN^high^ group was better. In the Progress Free Interval analysis of SKCM, the median survival time of the PTEN^low^ group was 29.6 (24.3-41). The median survival time of the PTEN^high^ group was 45.2 (36.4-53.9). Information on follow-up time or prognostic outcomes was missing in 14 samples. The result of the Log-rank test indicated that the difference in the distribution of survival time in the two groups was statistically significant (P = 0.04). The Cox regression result indicated that the difference in the distribution of the survival time in the two groups was statistically significant (P = 0.041). The results suggested that the prognosis of PTEN^high^ group was better.

1. **cBioPortal database analysis**

Firstly, “Skin” in the left option column in the cBioPortal database website (<https://www.cbioportal.org/>, version 4.1.15) (4) was selected. After “Skin Cutaneous Melanoma (TCGA, PanCancer Altas)” was checked in the “CUTANEOUS MELANOMA” group, the “Query By Gene” button was clicked. Secondly, “Mutations” and “Putative copy-number alterations from GISTIC” on the new page were checked, “PTEN” was entered in the “Enter Genes” box, “Submit Query” button was clicked to get the Distribution graph of PTEN genomic alterations in TCGA-SKCM data (Fig. 2E). After “mRNA vs CAN” button was clicked in the “Plots” option at the top of the page to get the “Putative copy-number alterations from GISTIC” graph (Fig. 2F). “Log2 copy-number values” from the “Copy Number Profile” option drop-down menu in the “Horizontal Axis” box on the left side of this page was selected, and then “Mutation Type”, “Structural Variant” and “Copy number” were removed at the top right of the page when checked. The “PTEN copy number and mRNA expression correlation plot” was obtained (Fig. 2G). The mutation status of CD69, IL7R and PTPRC were analyzed in the same way as PTEN (Figure. S6).

1. **The differential analysis of mRNA-miRNA-LncRNA**

The HT RNA-Seq-counts data from the TCGA SKCM project was downloaded. Data normalization and differential analysis of PTEN single genes were performed by the DESeq2 package (1.26.0 version) (5). The grouping of single-gene difference analysis was median grouping, PTEN^low^ group accounted for 50%, PTEN^high^ group accounted for 50%. Ensembl 101 (http://ftp.ensembl.org/pub/release-101/gtf/homo_sapiens/) (6) was used for definitions of some molecular types, please refer to Vega (http://vega.archive.ensembl.org/info/about/gene_and_transcript_types.html) (7). The results of the differential analysis showed that the total ID number of lncRNAs was 14077. Among them, there were 1457 lncRNAs satisfied the threshold of |log2(FC)|>0.5 and adjusted p <0.05. Under this threshold, there were 779 lncRNAs with high expression (logFC>0) in the PTEN^high^ group and 678 lncRNAs with low expression (logFC>0) in the PTEN^high^ group. These differential genes were imported into R and visualized by ggplot2 (version 3.3.3) (8) to obtain a volcano plot of PTEN single-gene differential analysis (Fig. 3A). These differential lncRNAs were sorted according to the |log2(FC)| value from large to small. After importing the top 15 differential lncRNAs into R, they can be visualized by ggplot2 (version 3.3.3) to get the visualization picture of the 15 lncRNAs most related to PTEN (Fig. 3D). The total ID number of miRNAs was 1495, of which 44 miRNAs met the threshold (|log2(FC)|>0.5 & adjusted p<0.05). Under this threshold, there were 33 miRNAs (logFC>0) highly expressed in the PTEN^high^ group, and 11 miRNAs (logFC<0) had low expression in the PTEN^high^ group. These differential miRNAs were imported into R and visualized by ggplot2 (version 3.3.3) to obtain a volcano plot (Fig. 3B) for PTEN single-gene differential analysis. Similarly, these differential miRNAs were sorted according to the |log2(FC)| value from large to small. After importing the top 15 differential miRNAs into R, they could be visualized by ggplot2 (version 3.3.3) (8) to get the visualization picture of the 15 miRNAs most related to PTEN (Fig. 3E). There were 3339 mRNAs met the threshold of |log2(FC)|>0.5 & adjusted p<0.05. Under this threshold, there were 1129 mRNAs with high expression (logFC>0) in the PTEN^high^ group, and 2210 mRNAs in the PTEN^high^ group with low expression (logFC<0). These differential mRNAs were imported into R and visualized by ggplot2 (version 3.3.3) (8) to obtain a volcano plot (Fig. 3C) for PTEN single-gene differential analysis. These differential mRNAs were sorted according to the |log2(FC)| value from large to small. After importing the top 15 differential mRNAs into R, they can be visualized by ggplot2 (version 3.3.3) (8) to get the visualization picture of the 15 mRNAs most related to PTEN (Fig. 3F).

1. **Cytoscape GO-KEGG analysis**

We chose the ClueGO plug-in (9) of Cytoscape (version 3.8.2) and selected “Functional Analysis” in the “Analysis Mode” box. Then “Homo Sapiens [9606]” in the “Load Marker List” box was selected, and symbol files of all lncRNAs, miRNAs and mRNAs that met |log2(FC)|>0.5 & adjusted p<0.05 difference were imported. The GO-KEGG enrichment analysis results could be obtained by checking “GO Biological Process”, “GO Cellular Component”, “GO Molecular Function” and “KEGG” in the “ClueGO Settings” box. Then “Show only Pathways with pV < 0.05” was checked. Enrichment analysis was performed when "Start" button was clicked (Fig. 3G-J).

## Construction of lncRNA-miRNA-mRNA triple regulatory network

Firstly, the 10 DEmiRNAs were used to predict related lncRNAs through starBase (<https://starbase.sysu.edu.cn/index.php>) (10). Then “miRNA-lncRNA” in the “miRNA-Target” drop-down menu was selected, “human” in the “Genome” option on the left menu was selected, and 10 miRNAs were entered one by one in the “microRNA” drop-down menu. The predicted lncRNAs can be seen in the “GENE NAME” project of “The miRNA-lncRNA interactions supported by Ago CLIP-seq DATA” box. 1272 predicted lncRNAs were obtained. The predicted lncRNAs and the 3464 DElncRNAs screened in the TCGA database were imported into R, and 404 lncRNAs were obtained by the Venndiagram package (version 1.6.0) (11) for an intersection. (Fig. 4A). The 404 lncRNAs were sorted according to the value of adjusted p, and the 10 lncRNAs with the largest differences were selected (FTX, ENTPD1-AS1, AL365361.1, OIP5-AS1, SNAI3-AS1, AL132656.2, PSMD6-AS2, MALAT1, POLR2J4, and WDFY3-AS2), which were used for the subsequent construction of the ceRNA network.

Secondly, 10 miRNAs were used to predict mRNAs by TarBase (<https://dianalab.e-ce.uth.gr/html/diana/web/index.php?r=tarbasev8>, version 8) (12). “Homo sapiens” in “Species” in the “Filters” menu on the left side of TarBase website was selected, and then 10 miRNAs were entered in the “miRNAs” box to get the predicted mRNAs in the “Gene name” column. 11114 mRNAs were obtained in totally. These predicted mRNAs and the top 1000 mRNAs with the most significant differences in the TCGA database were imported into R, and 266 mRNAs were obtained by using the Venndiagram package (version 1.6.0) (11) for intersection (Fig. 4B).

The predicted 266 mRNAs were sorted according to the value of adjusted P, and the top 50 mRNAs with the largest differences were imported into cytoHubba (13) for key gene prediction. Firstly, 50 mRNAs were imported into the “Multiple Protein by Names” column of the STRING (<https://cn.string-db.org/>, version 11.5) (14) website. “Homo sapiens” was selected in the “Organism” menu, and “search” was clicked to perform interaction analysis. In the “Export” option, the TSV format file of 50 mRNAs interacting with each other was downloaded. Secondly, the interaction file in TSV format was imported into the cytoHubba plug-in (13). In the “Import Network From Table” option, the genes in the left column are defined as “node1”, and the genes in the right column are defined as “node2”. Then, “Calculate” in “Nodes Scores” in the cytoHubb menu was selected, and “Top 10 nodes ranked by Degree” was selected in Hubba nodes option. The 10 Hub gene graph that had been screened out would be obtained after clicking “Submit” (Fig. 4C). The 3 mRNAs with the largest “Degree” values were the most meaningful Hub genes.

According to the method described above, 20 miRNAs corresponding to the 10 lncRNAs with the most significant differences were predicted through the starBase database (<https://starbase.sysu.edu.cn/>, version 2.0) (10). Then, the most relevant 8 miRNAs of the 3 Hubgenes were predicted by the TarBase database (12). The prediction results of the two parts were imported into R, the intersection was obtained by the Venndiagram package (version 1.6.0) (11), and the 6 miRNAs in common were obtained. After 10 lncRNAs, 6 miRNAs and 3 mRNAs were imported into the STRING database (version 11.5) for interaction analysis, and the interaction file in TSV format was downloaded. The interaction file was imported into the cytoHubba plug-in (13), and the final ceRNA network map was screened by the method described above (Fig. 4E). We imported 3 Hub genes into the STRING database (version 11.5). And “custom value” in the drop-down menu of the “max number of interactors to show” item was selected, and “100” was entered in “max interactors” to obtain a graph of related interacting genes that may be regulated by the ceRNA network (Fig. 4F). The related TSV format file was downloaded. The interaction file in the TSV format was imported into the Metascape database ([https://metascape.org/gp/index.html, version](https://metascape.org/gp/index.html,%20version) 3.0) (15). “H. sapiens” was selected from the “Input as species” and “Analysis as species” drop-down menus in “Step 2”. Then “Express Analysis” was clicked in “Step 3” to analyze. And the analysis report was downloaded to get the enrichment analysis result graph (Fig. G-K).

**7. The expression analysis of ceRNA-related genes in normal, primary SKCM and metastasis groups by UALCAN database**

Firstly, “TCGA” in the upper item column on the website of the UALCAN database (<http://ualcan.path.uab.edu/>) (16) was clicked. Secondly, “CD69” was entered in the “Enter gene symbol” box, and then “TCGA dataset” drop-down menu was clicked and “Skin cutaneous melanoma” was selected. Thirdly, “Explore” was clicked to analyze. “Expression” in the “Links for analysis” option was clicked to get the histogram of “Expression of CD69 in SKCM based on Sample types” (Fig.5A). “Individual cancer stages” from the drop-down menu of “CD69 expression profile based on” was selected to get a histogram of “CD69 expression profile based on Individual cancer stages” (Fig. 5I). The remaining lncRNAs, miRNAs and mRNAs in the ceRNA network were analyzed according to the above method (Fig. 5B-H and Fig. 5J-P).

**8. ceRNA network (lncRNAs, miRNAs and mRNAs) and SKCM overall survival analysis**

The FPKM data from HT RNA-Seq were downloaded from the TCGA (<https://portal.gdc.cancer.gov/>, version 1.28.0) SKCM project, then it was converted into TPM data, and performed log2 transformation. After the processed data were imported into R, the survival data were statistically analyzed by the survival package (version 3.2.10) (2). The selected prognosis type was Overall Survival, and the statistical results were visualized by the survminer package (version 0.4.9) (3) (Fig 2D). The median grouping method was used to analyze the Overall Survival of SKCM. There were 227 cases in the CD69^low^ group, with a median survival time of 55.2 (47.6-69). There were 229 cases in the CD69^high^ group, with a median survival time of 150.2 (104.5-179). Information on follow-up time or prognostic outcomes was missing in 15 samples. The results of Log-rank test showed that the difference in survival time distribution between the two groups was statistically significant (P<0.001). The Cox regression result showed that the difference in survival time distribution of the two groups was statistically significant (P<0.001). The results suggested that the CD69^high^ group had a better prognosis (Fig. 6A). The remaining lncRNAs, miRNAs and mRNAs in the ceRNA network were analyzed according to the above method, and the corresponding survival curves could be obtained (Fig. 6B-K).

**9. Cellular localization of lncRNAs**

We entered MALAT1 in the "Search" column on the homepage of LNCipedia (<http://lncipedia.org/>, version 5.2) (17). “MALAT1:1” in the search results was chosen, and the RNA sequence of MALAT1 in the “RNA sequence” box was copied. Next, the copied RNA sequence was pasted into the “Upload lncRNA sequence” box on the lncLocator (<http://www.csbio.sjtu.edu.cn/bioinf/lncLocator/>, version 1.0) (18) page, and the “Submit” button was clicked to query. The analysis results were presented in the form of a histogram (Fig.7A). The cellular localization of OIP5-AS1 was also analyzed by the same method described above.

**10. Correlation analysis between lncRNAs and miRNAs, miRNAs and mRNAs in the ceRNA network**

The level 3 HTSeq-FPKM data from RNAseq and BCGSC miRNA Profiling data from miRNAseq were downloaded in TCGA (<https://portal.gdc.cancer.gov/>, version 1.28.0) SKCM project. The data were divided into two groups, hsa-miR-374b-5p and MALAT1. The results of the Shapiro-Wilk normality test showed that the variables did not meet the normal distribution (P < 0.05). As a result, we calculated the Spearman correlation coefficient. Correlation analysis results of hsa-miR-374b-5p and MALAT1 showed Pearson correlation coefficient r was 0.162 (P < 0.001). Spearman correlation coefficient r was 0.117 (P=0.013). It indicates that there is a positive correlation between hsa-miR-374b-5p and MALAT1. The analysis result was visualized by the ggplot2 (version 3.3.3) (8) package in R, and a scatter plot of the correlation between hsa-miR-374b-5p and MALAT1 was obtained (Fig. 7E). The correlation analysis between the remaining lncRNAs and miRNAs or miRNAs and mRNAs was analyzed according to the above method, and the corresponding correlation scatter plots (Fig. 7F-H) were obtained.

**11. Gene methylation analysis (*IL7R*, *CD69*, and *PTPRC*)**

The “Analyze” item in the horizontal column on the official website of DiseaseMeth (<http://bio-bigdata.hrbmu.edu.cn/diseasemeth/>, version 2.0) (19) was chosen. “Skin Cutaneous Melanoma” in the “Disease” option was checked, and “CD69” was entered into the “Gene Symbol/ Transcript ID/ Genomic_Interval” project. “Array-based technology” in the “Technology Experimental Platform” project was checked, and “Analysis” button was clicked to analyze. “NM_001781” in the “uniMatched” box was chosen and “Analysis” button was clicked to continue the analysis. On the new page, “chr12:9912997-9915497” was checked to continue the analysis, and the histogram of CD69 methylation levels in normal tissue and SKCM tissue could be obtained (Fig. 8A).

"TCGA" in the upper item column on the website of the UALCAN database (<http://ualcan.path.uab.edu/>) (16) was chosen, and “CD69” was entered in the “Enter gene symbol” box. In the “TCGA dataset” drop-down menu, “Skin cutaneous melanoma” was selected, and “Explore” button was clicked for analysis. “Methylation” in the “Links for analysis” option was chosen to get a histogram of “Promoter methylation level of CD69 in SKCM” (Fig.8B). Next, “Skin Cutaneous Melanoma [SKCM] TCGA March 2017” in the drop-down menu of “TCGA cancer datasets” on the Methsurv's official website (<https://biit.cs.ut.ee/methsurv/>) (20) was selected. “CD69” was filled in the “Gene” option and “Open_Sea” was selected in the “Relation to island” option. “TSS1500” was selected in the “Genomic Region” option, “cg25769852” was selected in the “CpG site” option, and “best” was selected in the “Split by” option. After that, the website presented the analysis result graph by itself. Then “Single CpG” button was clicked to get Figure 8G, J and M. “Gene visualization” button was clicked to get Figure 8S. “CD69” was entered in the “Enter a gene or miRNA name” box on the website of the MEXPRESS database (<http://mexpress.be>, version new) (21). “SKCM” was selected in the “And select a cancer type” drop-down menu, and “PLOT” was clicked to analyze. The methylation graph of CD69 was obtained (Fig. 8P). The methylation analysis of IL7R and PTPRC was performed as above.

**12. Correlation analysis of ceRNA and immune infiltration in SKCM**

We selected “SCNA” in the upper item bar on the official website of the TIMER database (<https://cistrome.shinyapps.io/timer/>, version 2.0) (22). “SKCM” in the drop-down menu of “Cancer Type” was selected. “CD69” was entered in the “Gene Symbol” box, and “Submit” button was clicked to get the program of the relationship between the gene copy number of CD69 and the level of immune cell infiltration (Fig. 9A).

Next, “Gene” in the upper item bar was selected and “CD69” was entered into the “Gene Symbol” item. “SKCM” was selected in the drop-down menu of “Cancer Type”, “Submit” button was clicked to obtain the correlation program between CD69 expression and SKCM immune infiltration level (Fig. 9D).

“Survival” in the upper item bar was chosen, then “SKCM” was selected in the drop-down menu of “Cancer Type”. “CD69” was entered into the “Gene Symbol” item, and “Plot KM Curve” button was clicked to obtain a graph of the relationship between immune infiltration and overall survival in SKCM (Fig. 9G). The analysis methods of IL7R and PTPRC referred to the method above (Fig. 9B-C, E-F).

**13. Single-gene logistic regression of OIP5-AS1, MALAT1, CD69, IL7R and PTPRC**

The level 3 FPKM data from HT RNA-Seq were downloaded from the TCGA (<https://portal.gdc.cancer.gov/>, version 1.28.0) SKCM project, then it was converted into TPM data, and performed log2 transformation. The independent variable was CD69, and the independent variable type was Low/High binary variable. Then the downloaded data was imported into R, and single-gene logistic regression on the data was performed through the basic package of R. The single-gene logistic regression analysis method for OIP5-AS1, MALAT1 IL7R and PTPRC was performed as described above (Table. SI 2-6).

**14. Univariate and Multivariate Cox Regression Analysis**

The level 3 FPKM data from HT RNA-Seq were downloaded from the TCGA (https://portal.gdc.cancer.gov/,version 1.28.0) SKCM project, then it was converted into TPM data, and performed log2 transformation. After the processed data were imported into R (version 3.6.3), the survival data were statistically analyzed by the survival package (version 3.2.10) (2). The Cox regression module was used for analysis, and the prognosis type was Overall Survival. Included variables were PTEN; IL7R; PTPRC; CD69; MALAT1; OIP5-AS1; T stage; N stage; M stage; Pathologic stage; Gender; Radiation therapy; Race; Age; Weight; Height; BMI; Breslow depth; Tumor tissue site; Melanoma ulceration; Melanoma Clark level (Table. SI 7-12).

**15. The expression of PTEN in normal and tumor tissues**

The TPM data from RNAseq in TCGA and GTEx was unified processed through Toil process by UCSC XENA ([https://xenabrowser.net/datapages/](https://xenabrowser.net/datapages/，version)) (23). The SKCM data in TCGA and corresponding normal tissue data in GTEx were extracted. The TPM data from RNAseq were log2 transformed for expression comparison between samples. Shapiro-Wilk normality test result showed that the samples did not meet the normality test (P < 0.05). It was recommended to choose the Mann-Whitney U test (Wilcoxon rank sum test). The results of Mann-Whitney U test (Wilcoxon rank sum test) showed that the expression level of PTEN in the Tumor group was significantly lower than that in the Normal group. The median difference between the two groups was -0.393 (-0.477 - -0.31), and the difference was statistically significant (P < 0.001). The analysis results were visualized by ggplot2 (version 3.3.3) (8) package (Fig. S1).

**16. DElncRNAs, DEmiRNAs, and DEmRNAs between SKCM samples and adjacent non-tumor samples**

The level 3 FPKM data from HT RNA-Seq were downloaded from the TCGA (https://portal.gdc.cancer.gov/,version 1.28.0) SKCM project. After the downloaded data were imported into R (version 3.6.3), the data difference analysis was performed by DESeq2 (version 1.26.0) (5). The analysis results were classified by DElncRNAs, DEmiRNAs, and DEmRNAs and visualized by ggplot2 (version 3.3.3) (8) package (Fig. S2). The screening criteria was |log2(FC)|>1 & adjusted p<0.05.

**17. Expression profile of ten hub genes in samples with different PTEN expression levels**

The level 3 HTSeq-FPKM data of RNAseq and miRNAseq data of BCGSC miRNA Profiling were downloaded in TCGA (<https://portal.gdc.cancer.gov/>, version 1.28.0) SKCM project. The data were divided into two groups, hsa-miR-374b-5p and PTEN. The results of the Shapiro-Wilk normality test showed that there were variables that did not meet the normal distribution (P < 0.05). Then the Spearman correlation coefficient was calculated. The analysis results were then visualized by the ggplot2 (version 3.3.3) (8) package in R, and a scatter plot of the correlation between hsa-miR-374b-5p and PTEN was obtained (Fig. S3). The correlation analysis between lncRNAs, miRNAs, miRNAs and PTEN were analyzed according to the above method (Fig. S3).

**18. The expression value of hub DElncRNAs (OIP5-AS1 and MALAT1), DEmRNAs (PTPRC, IL7R and CD69) in SKCM and non-tumor samples**

The TPM data of RNAseq in TCGA and GTEx were unified processed through Toil process by UCSC XENA (<https://xenabrowser.net/datapages/>) (23). The SKCM data in TCGA and corresponding normal tissue data in GTEx were extracted. Then, the extracted data were log2 transformed to compare OIP5-AS1 expression between samples. The downloaded data were imported into R for analysis. The Shapiro-Wilk normality test result showed that the sample did not meet the normality test (P < 0.05). Therefore, the Mann-Whitney U test (Wilcoxon rank sum test) was used to analyze the data. Result showed that the expression of OIP5-AS1 in Tumor tissues was higher than in Normal tissues. The median difference between the two groups was 0.091 (0.028 - 0.153), and the difference was statistically significant (P = 0.005). The analysis method of differences in expression of MALAT1, PTPRC, IL7R and CD69 between SKCM tumor tissues and normal tissues were the same as OIP5-AS. (Fig. S4A).

To further validate our results, we downloaded the gene expression datasets of GSE3189 (including 45 SKCM samples and seven normal skin samples; platform, GPL96) from the GEO database (<https://www.ncbi.nlm.nih.gov/geo/>). The downloaded data were imported into R for Difference analysis by DESeq2 (1.26.0 version) (5) package. |log2(FC)|>0.5 & adjusted p<0.05 was used as the standard to screen for differential genes. And labeled PTPRC, IL7R and CD69, the analysis result was visualized by ggplot2 (version 3.3.3) (8) package. PTPRC, IL7R and CD69 were labeled on the volcano map (Fig. S4B).

**19. The expression levels of three differential mRNAs (IL7R, CD69 and PTPRC) in different cancer cell lines of human organs**

We entered IL7R in the search box on the website of the CCLE database (<https://portals.broadinstitute.org/ccle/>) (24). Then “Characterization” in the upper horizontal item column was selected to get the expression levels of IL7R in different cancer cell lines of human organs. CD69 and PTPRC were analyzed in the same way as IL7R (Fig. S5).

**20. Functional enrichment analysis of 2 DElncRNAs and 3 DEmRNAs**

The level 3 FPKM data from HT RNA-Seq were downloaded from the TCGA (https://portal.gdc.cancer.gov/,version 1.28.0) SKCM project, then it was converted into TPM data, and performed log2 transformation. The data was imported into R for group sorting. The counts format data of SKCM was downloaded from TCGA. The DESeq2 (1.26.0 version) (5) package was used to perform a CD69 difference analysis between the two groups. Then, the enrichment analysis result was performed through the cluster Profiler (version 3.14.3) (25) package. The results of the enrichment analysis were visualized by the ggplot2 (version 3.3.3) (8) package. The single-gene enrichment analysis method of OIP-AS1, MALAT1, IL7R and PTPRC was the same as that of CD69 (Fig. S7).

**References**

1. Sjöstedt E, Zhong W, Fagerberg L, Karlsson M, Mitsios N, Adori C, et al. An atlas of the protein-coding genes in the human, pig, and mouse brain. Science (New York, NY). 2020;367(6482).

2. Rizvi AA, Karaesmen E, Morgan M, Preus L, Wang J, Sovic M, et al. gwasurvivr: an R package for genome-wide survival analysis. Bioinformatics (Oxford, England). 2019;35(11):1968-70.

3. Groeneveld CS, Chagas VS, Jones SJM, Robertson AG, Ponder BAJ, Meyer KB, et al. RTNsurvival: an R/Bioconductor package for regulatory network survival analysis. Bioinformatics (Oxford, England). 2019;35(21):4488-9.

4. Cerami E, Gao J, Dogrusoz U, Gross BE, Sumer SO, Aksoy BA, et al. The cBio cancer genomics portal: an open platform for exploring multidimensional cancer genomics data. Cancer discovery. 2012;2(5):401-4.

5. Love MI, Huber W, Anders S. Moderated estimation of fold change and dispersion for RNA-seq data with DESeq2. Genome biology. 2014;15(12):550.

6. Hunt SE, McLaren W, Gil L, Thormann A, Schuilenburg H, Sheppard D, et al. Ensembl variation resources. Database. 2018;2018.

7. Harrow JL, Steward CA, Frankish A, Gilbert JG, Gonzalez JM, Loveland JE, et al. The Vertebrate Genome Annotation browser 10 years on. Nucleic acids research. 2014;42(Database issue):D771-9.

8. Wilkinson L. ggplot2: Elegant Graphics for Data Analysis by WICKHAM, H. 2011;67(2):678-9.

9. Bindea G, Mlecnik B, Hackl H, Charoentong P, Tosolini M, Kirilovsky A, et al. ClueGO: a Cytoscape plug-in to decipher functionally grouped gene ontology and pathway annotation networks. Bioinformatics (Oxford, England). 2009;25(8):1091-3.

10. Li JH, Liu S, Zhou H, Qu LH, Yang JH. starBase v2.0: decoding miRNA-ceRNA, miRNA-ncRNA and protein-RNA interaction networks from large-scale CLIP-Seq data. Nucleic acids research. 2014;42(Database issue):D92-7.

11. Chen H, Boutros PC. VennDiagram: a package for the generation of highly-customizable Venn and Euler diagrams in R. BMC bioinformatics. 2011;12:35.

12. Karagkouni D, Paraskevopoulou MD, Chatzopoulos S, Vlachos IS, Tastsoglou S, Kanellos I, et al. DIANA-TarBase v8: a decade-long collection of experimentally supported miRNA-gene interactions. Nucleic acids research. 2018;46(D1):D239-d45.

13. Chin CH, Chen SH, Wu HH, Ho CW, Ko MT, Lin CY. cytoHubba: identifying hub objects and sub-networks from complex interactome. BMC systems biology. 2014;8 Suppl 4(Suppl 4):S11.

14. Szklarczyk D, Gable AL, Nastou KC, Lyon D, Kirsch R, Pyysalo S, et al. The STRING database in 2021: customizable protein-protein networks, and functional characterization of user-uploaded gene/measurement sets. Nucleic acids research. 2021;49(D1):D605-d12.

15. Zhou Y, Zhou B, Pache L, Chang M, Khodabakhshi AH, Tanaseichuk O, et al. Metascape provides a biologist-oriented resource for the analysis of systems-level datasets. Nature communications. 2019;10(1):1523.

16. Chandrashekar DS, Bashel B, Balasubramanya SAH, Creighton CJ, Ponce-Rodriguez I, Chakravarthi B, et al. UALCAN: A Portal for Facilitating Tumor Subgroup Gene Expression and Survival Analyses. Neoplasia (New York, NY). 2017;19(8):649-58.

17. Volders PJ, Anckaert J, Verheggen K, Nuytens J, Martens L, Mestdagh P, et al. LNCipedia 5: towards a reference set of human long non-coding RNAs. Nucleic acids research. 2019;47(D1):D135-d9.

18. Cao Z, Pan X, Yang Y, Huang Y, Shen HB. The lncLocator: a subcellular localization predictor for long non-coding RNAs based on a stacked ensemble classifier. Bioinformatics (Oxford, England). 2018;34(13):2185-94.

19. Lv J, Liu H, Su J, Wu X, Liu H, Li B, et al. DiseaseMeth: a human disease methylation database. Nucleic acids research. 2012;40(Database issue):D1030-5.

20. Modhukur V, Iljasenko T, Metsalu T, Lokk K, Laisk-Podar T, Vilo J. MethSurv: a web tool to perform multivariable survival analysis using DNA methylation data. Epigenomics. 2018;10(3):277-88.

21. Koch A, De Meyer T, Jeschke J, Van Criekinge W. MEXPRESS: visualizing expression, DNA methylation and clinical TCGA data. BMC genomics. 2015;16(1):636.

22. Li T, Fan J, Wang B, Traugh N, Chen Q, Liu JS, et al. TIMER: A Web Server for Comprehensive Analysis of Tumor-Infiltrating Immune Cells. Cancer research. 2017;77(21):e108-e10.

23. Vivian J, Rao AA, Nothaft FA, Ketchum C, Armstrong J, Novak A, et al. Toil enables reproducible, open source, big biomedical data analyses. Nature biotechnology. 2017;35(4):314-6.

24. Barretina J, Caponigro G, Stransky N, Venkatesan K, Margolin AA, Kim S, et al. The Cancer Cell Line Encyclopedia enables predictive modelling of anticancer drug sensitivity. Nature. 2012;483(7391):603-7.

25. Kolberg L, Raudvere U, Kuzmin I, Vilo J, Peterson H. gprofiler2 -- an R package for gene list functional enrichment analysis and namespace conversion toolset g:Profiler. F1000Research. 2020;9.
