# Supplementary material for: Integrated analysis to reveal potential therapeutic targets and prognostic biomarkers of skin cutaneous melanoma
Source: Front Immunol. 2022 Aug 11;13:914108. doi: 10.3389/fimmu.2022.914108 (PMC9402985; doi:10.3389/fimmu.2022.914108)
Supplement: Supplementary file 3 [file DataSheet_3.pdf]

Supplemental Figure 1

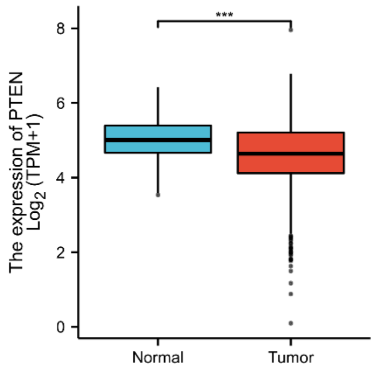

Fig. S1. The expression of PTEN in normal and tumor tissues separately, analyzed by TCGA and GTEx databases. *$p < 0.05$, **$p < 0.01$, ***$p < 0.001$.

Supplemental Figure 2

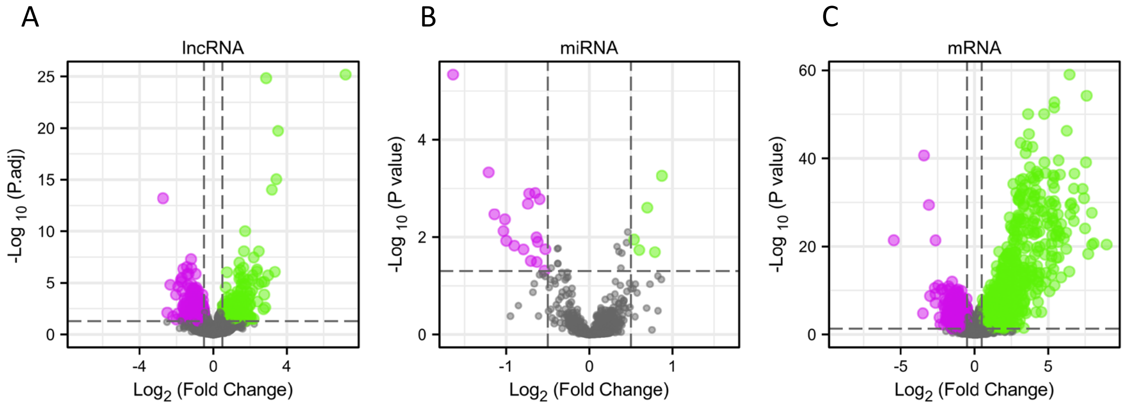

Fig. S2. Volcano plots of DElncRNAs, DEmiRNAs, and DEmRNAs between SKCM samples and adjacent non-tumor samples. Green indicates upregulated genes, and purple indicates downregulated genes. The volcano plots described (A) 584DElncRNAs (|log2Fold- Change| > 0.5 and adjusted p value < 0.05), among them, there are 226 highly expressed lncRNAs and 358 lowly expressed lncRNAs. (B) 23DEmiRNAs (|log2FoldChange|>0.5 and adjusted p value < 0.05), among them, there are 5 with high expression and 18 with low expression. (C) 3102DEmRNAs (| log2FoldChange| > 0.5 and adjusted p value < 0.05). Among them, 1510 genes are highly expressed, and 1592 genes are lowly expressed.

Supplemental Figure 3

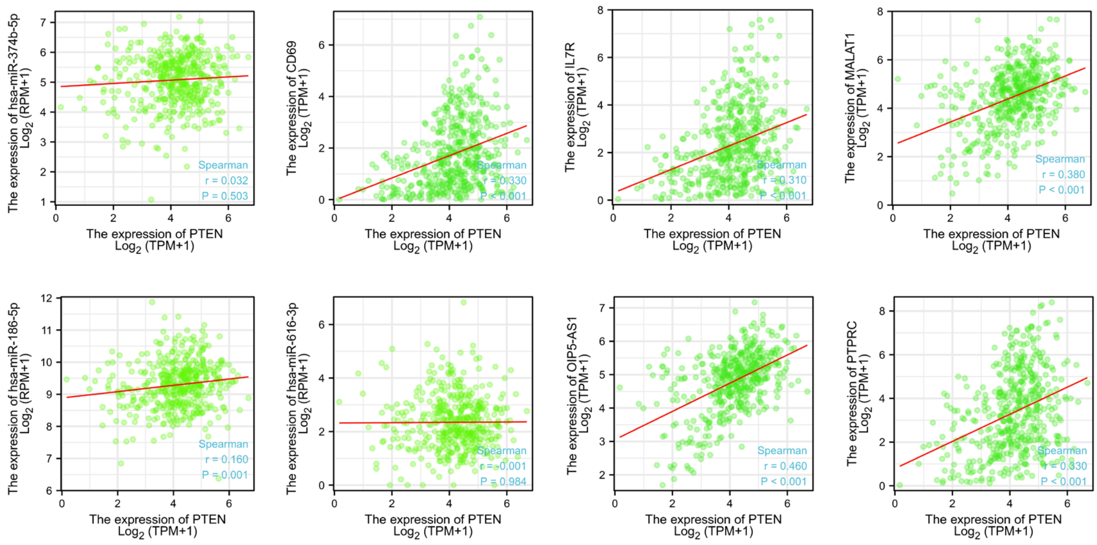

Fig. S3. Expression profile of ten hub genes in samples with different PTEN expression levels.

Supplemental Figure 4

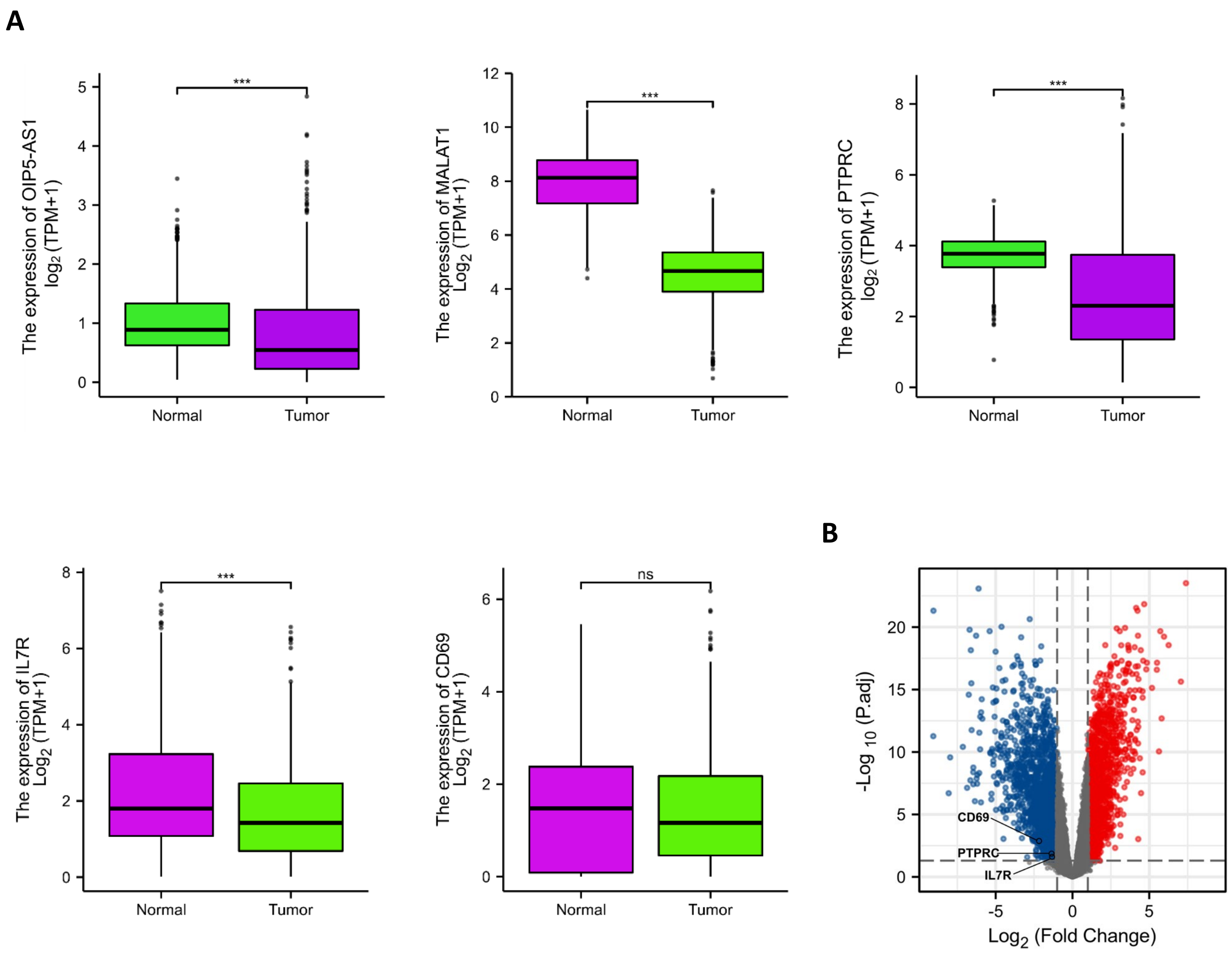

Fig. S4. (A) The expression value of hub DElncRNAs (OIP5-AS1 and MALAT1), DEmRNAs (PTPRC, IL7R and CD69) in SKCM and non-tumor samples respectively. (B) The expression value of DEmRNAs (PTPRC, IL7R and CD69) in SKCM and non-tumor samples of GEO dataset. *p < 0.05, **p < 0.01, ***p < 0.001.

Supplemental Figure 5

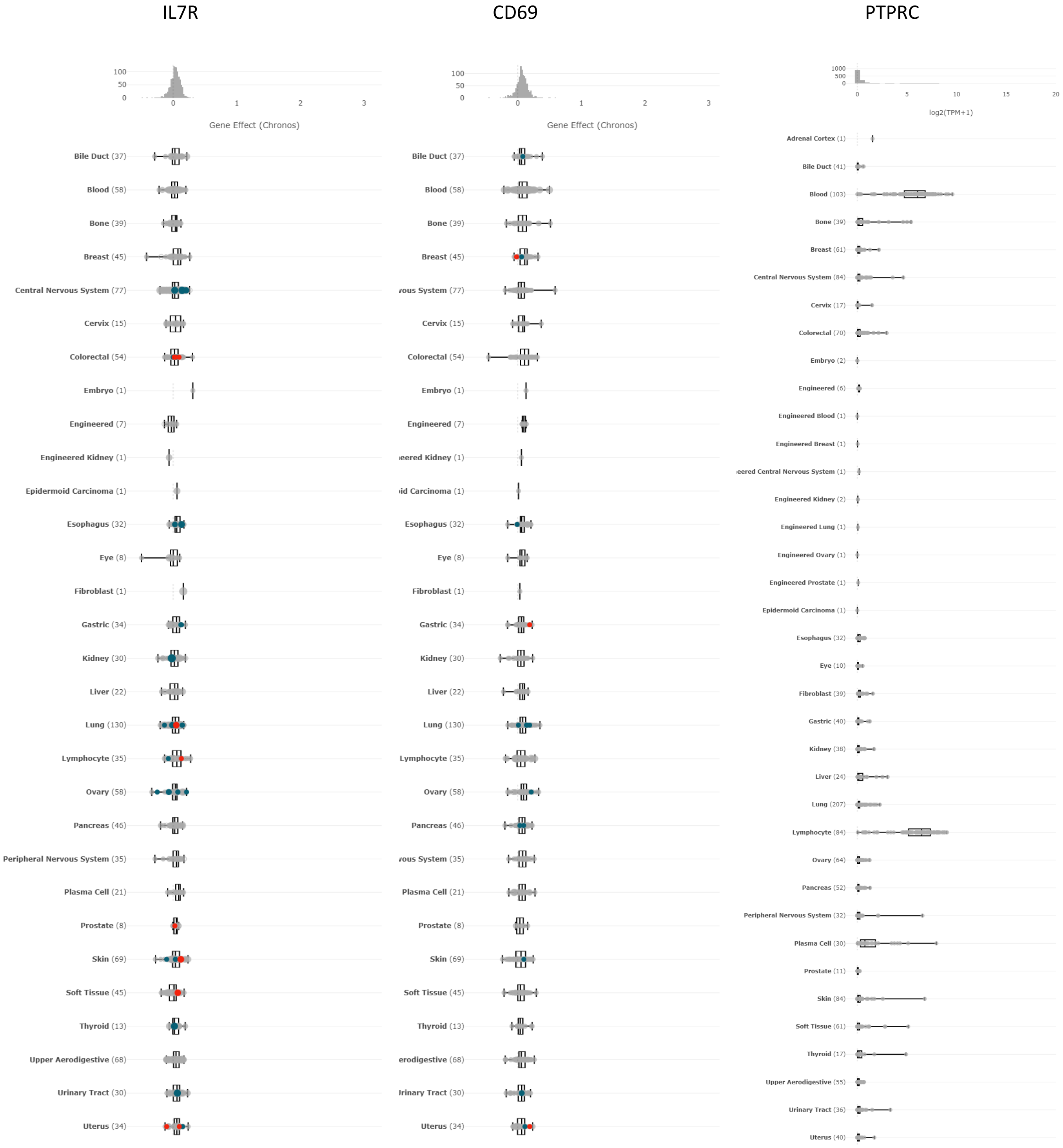

Fig. S5. The expression levels of three differential mRNAs (IL7R, CD69 and PTPRC) in different cancer cell lines of human organs.

Supplemental Figure 6

A

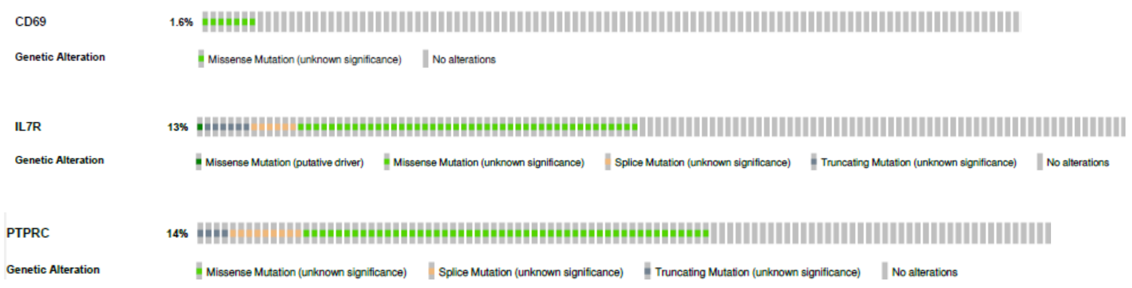

B

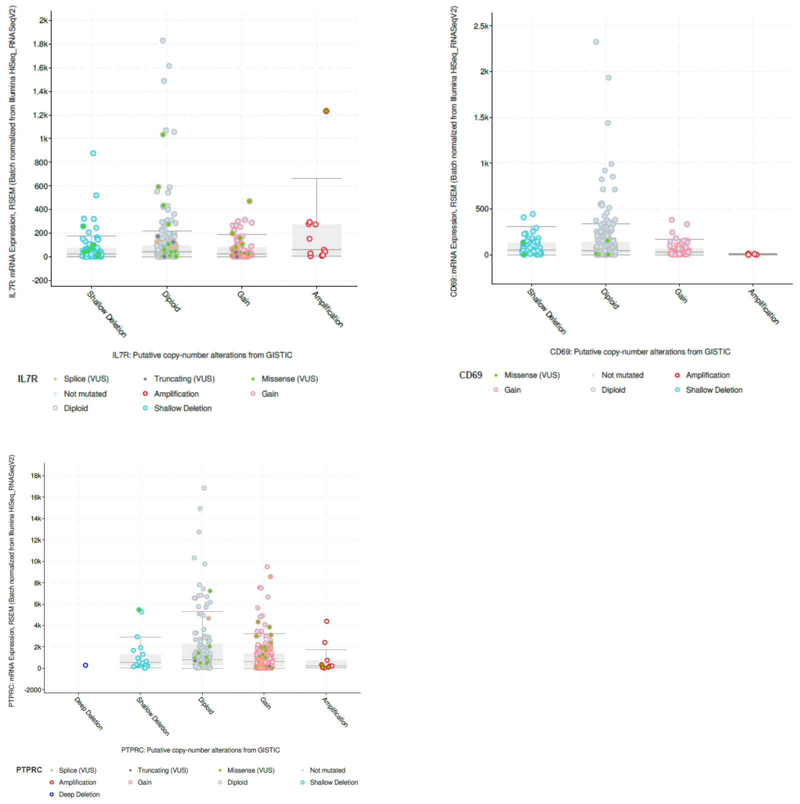

Fig. S6. The mutation status of CD69, IL7R and PTPRC in SKCM. (A) The distribution of CD69, IL7R and PTPRC genomic alterations of SKCM in the TCGA dataset is shown on the cBioPortal OncoPrint plot. The correlation analysis between CD69, IL7R and PTPRC copy number and mRNA expression are shown in point plot (B).

Supplemental Figure 7

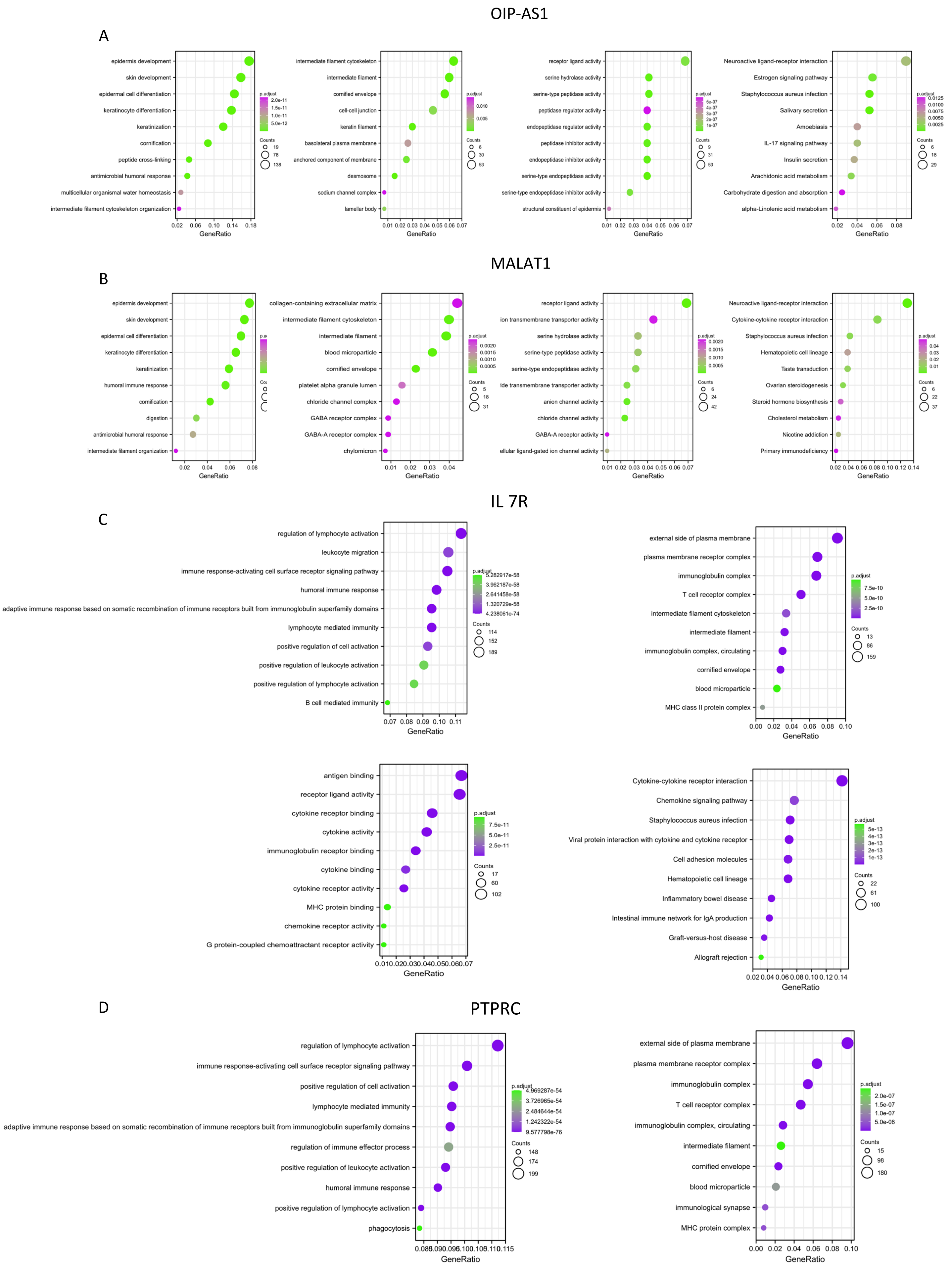

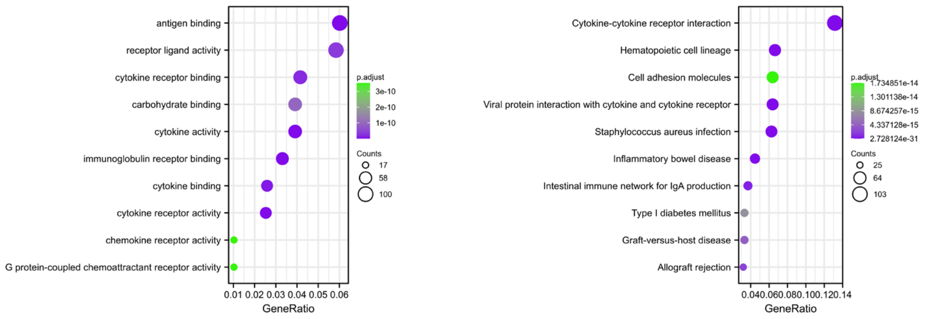

E

CD69

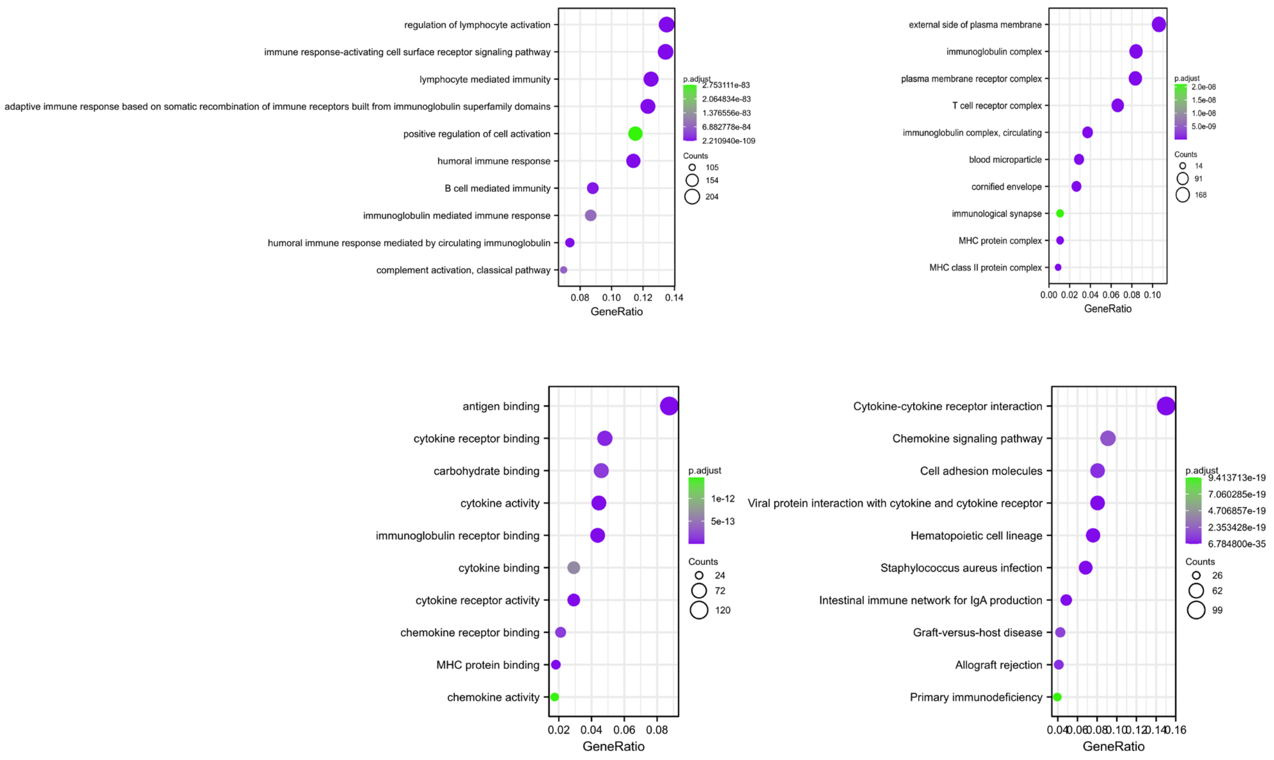

Fig. S7. Functional enrichment analysis of 2 DElncRNAs and 3 DEmRNAs. Functional enrichment analysis (including GO and KEGG) of OIP-AS1, MALAT1, IL7R and PTPRC, CD69 associated genes in SKCM.
